# Supplementary material for: Shining a light on camouflage evolution: Using genetic algorithms to determine the effects of geometry and lighting on optimal camouflage
Source: PLoS One. 2026 Apr 29;21(4):e0346231. doi: 10.1371/journal.pone.0346231 (PMC13127904; doi:10.1371/journal.pone.0346231)
Supplement: S2 File — Contains the full stats tables for all of the analyses included in the main text. (PDF) [file pone.0346231.s002.pdf]

Supplementary Statistics for Hancock et al. "Shining a light on camouflage evolution: using genetic algorithms to determine the effects of geometry and lighting on optimal camouflage."

George R.A. Hancock, [1]* Innes C. Cuthill[2], Jolyon Troscianko[1]

*Corresponding author: ghancockzoology@gmail.com

1. Centre for Ecology & Conservation, University of Exeter, Penryn, TR10 9FE, UK

2. School of Biological Sciences, University of Bristol, Tyndall Avenue, Bristol BS8 1TQ, UK

Background Variation and Fitness

Table S2.1. Stats output for the effect of global background variation in the luminance L*), green-red (a*), blue-yellow (b*) and depth (Z) on fitness and the evolution of increased fitness. The lower the effect size the redder the ***β*** value.

	Effect					Effect : Generation				
Fixed Effect	β	SE	SD	t	p value	β	SE	SD	t	p value
L	0.027	0.00182	42330	15.000	<0.001	0.001	0.000155	42330	5.767	<0.001
A	-0.011	0.001852	42330	-5.737	<0.001	0.002	0.000157	42330	13.142	<0.001
B	0.002	0.001851	42330	1.088	0.2764	0.002	0.000156	42330	14.241	<0.001
Z	-0.003	0.001842	42330	-1.760	0.0784	0.002	0.000157	42330	11.400	<0.001
LA	0.034	0.000978	42190	35.100	<0.001	0.013	0.00094	42330	13.500	<0.001
LB	0.018	0.001831	42330	9.823	<0.001	0.002	0.000155	42330	11.786	<0.001
LZ	0.019	0.001821	42330	10.300	<0.001	0.002	0.000155	42330	12.760	<0.001
AB	-0.005	0.001854	42330	-2.494	0.0126	0.002	0.000157	42330	15.081	<0.001
AZ	-0.008	0.001847	42330	-4.268	<0.001	0.002	0.000157	42330	15.081	<0.001
BZ	-0.001	0.001846	42330	-0.296	0.7673	0.002	0.000156	42330	15.322	<0.001
LAB	0.010	0.001841	42320	5.175	<0.001	0.002	0.000155	42330	14.613	<0.001
LAZ	0.009	0.001835	42330	4.646	<0.001	0.002	0.000155	42330	15.740	<0.001
LBZ	0.014	0.001832	42320	7.396	<0.001	0.002	0.000155	42330	14.814	<0.001
ABZ	-0.005	0.00185	42330	-2.442	0.0146	0.002	0.000157	42330	15.809	<0.001
LABZ	0.007	0.00184	42320	3.797	<0.001	0.002	0.000155	42330	16.067	<0.001

Evolution and Fitness (Survival Time)

Lighting Treatments

Table S2.2. Stats output for the effect of the number of generations, lighting and background 3D variation on fitness, excluding outliers. Linear mixed model formula:

lmer(log(SurvivalTime) ~ Generation * Light * $3D_{Variation}$ + (1|Habitat) + (1|Population) …

	Predictor	β	SE	DF	t	p value
log Fitness (Capture Time)	Intercept	0.471	0.02	35	29.43	<0.001
	Generation	0.050	0.00	36210	31.08	<0.001
	Mixed	-0.024	0.01	41	-2.35	0.024
	Diffuse	-0.020	0.01	42	-1.84	0.072
	Mean 3D Var	0.033	0.02	33	1.97	0.057
	Gen : Mixed	-0.009	0.00	36210	-4.17	<0.001
	Gen : Diffuse	-0.004	0.00	36210	-1.90	0.058
	Gen : 3D	0.009	0.00	36210	5.90	0.000
	Mixed : 3D	-0.022	0.01	41	-2.10	0.042
	Diffuse : 3D	-0.025	0.01	41	-2.43	0.020
	Gen : Mixed : 3D	-0.002	0.00	36210	-0.72	0.470
	Gen : Diffuse : 3D	0.005	0.00	36210	2.26	0.024

Mixed Lighting

Table S2.3. Stats output for the effect of the number of generations, background lighting and background 3D variation on fitness for mixed lighting populations, excluding outliers. Linear mixed model formula:

lmer(log(SurvivalTime) ~ Generation * Light * $3D_{Variation}$ + (1|Habitat) + (1|Population) …

	Predictor	β	SE	DF	t	p value
log Fitness (Capture Time)	Intercept	0.456	0.02	22	27.22	<0.001
	Generation	0.040	0.00	12070	17.69	<0.001
	bg Diffuse	-0.014	0.00	12070	-4.33	<0.001
	3D	0.014	0.02	22	0.85	0.404
	Gen : Diffuse	0.001	0.00	12070	0.25	0.806
	Gen : 3D	0.011	0.00	12070	4.91	0.000
	Diffuse: 3D	-0.011	0.00	12070	-3.39	0.001
	Gen : Diffuse : 3D	-0.007	0.00	12070	-2.11	0.035

Camouflage Metrics and Fitness (Survival Time)

Luminance Difference

Table S2.4. Stats output for the effect of luminance match, lighting and background 3D variation on fitness, excluding outliers. Linear mixed model formula:

lmer(log(SurvivalTime) ~ $Luminance_{Difference}$ * Light * $3D_{Variation}$ + (1|Habitat) + (1|Population) …

	Predictor	β	SE	DF	t	p value
log Fitness (Capture Time)	Intercept	0.470	0.02	34	29.61	<0.001
	L Difference	-0.036	0.00	35780	-20.21	<0.001
	Mixed	-0.022	0.01	41	-2.19	0.034
	Diffuse	-0.018	0.01	42	-1.70	0.096
	Mean 3D Var	0.028	0.02	33	1.72	0.095
	LD : Mixed	0.003	0.00	35550	1.07	0.285
	LD : Diffuse	0.007	0.00	35910	2.75	0.006
	LD : 3D	-0.011	0.00	36250	-5.89	0.000
	Mixed : 3D	-0.019	0.01	41	-1.89	0.065
	Diffuse : 3D	-0.020	0.01	41	-1.94	0.060
	LD : Mixed : 3D	0.001	0.00	36250	0.41	0.683
	LD : Diffuse : 3D	0.003	0.00	36230	1.04	0.301

Colour Difference

Table S2.5. Stats output for the effect of colour difference (Euclidean distance of a* and b*), lighting and background 3D variation on fitness, excluding outliers. Linear mixed model formula:

lmer(log(SurvivalTime) ~ $Colour_{Difference}$ * Light * $3D_{Variation}$ + (1|Habitat) + (1|Population) …

	Predictor	β	SE	DF	t	p value
log Fitness (Capture Time)	Intercept	0.468	0.01	36	31.49	<0.001
	AB Difference	-0.043	0.00	35500	-23.53	<0.001
	Mixed	-0.020	0.01	41	-1.99	0.053
	Diffuse	-0.014	0.01	42	-1.26	0.215
	Mean 3D Var	0.031	0.02	34	2.00	0.054
	ABD : Mixed	0.004	0.00	35470	1.47	0.142
	ABD : Diffuse	0.005	0.00	35780	2.19	0.028
	ABD : 3D	-0.008	0.00	35940	-4.96	<0.001
	Mixed : 3D	-0.018	0.01	41	-1.76	0.087
	Diffuse : 3D	-0.021	0.01	41	-2.05	0.046
	ABD : Mixed : 3D	0.008	0.00	35910	3.12	0.002
	ABD : Diffuse : 3D	0.004	0.00	36140	1.91	0.056

Pattern Contrast Difference

Table S2.6. Stats output for the effect of pattern difference (difference in L* StdDev), lighting and background 3D variation on fitness, excluding outliers. Linear mixed model formula:

lmer(log(SurvivalTime) ~ $Pattern_{Difference}$ * Light * $3D_{Variation}$ + (1|Habitat) + (1|Population) …

	Predictor	β	SE	DF	t	p value
log Fitness (Capture Time)	Intercept	0.478	0.02	35	29.89	<0.001
	Pattern Difference	-0.036	0.00	34420	-18.66	<0.001
	Mixed	-0.031	0.01	42	-2.95	0.005
	Diffuse	-0.043	0.01	44	-3.85	<0.001
	Mean 3D Var	0.020	0.02	33	1.23	0.227
	PD : Mixed	0.023	0.00	34240	8.58	<0.001
	PD : Diffuse	0.007	0.00	34930	2.21	0.027
	PD : 3D	-0.020	0.00	34980	-9.13	<0.001
	Mixed : 3D	-0.010	0.01	41	-0.92	0.361
	Diffuse : 3D	-0.026	0.01	44	-2.46	0.018
	PD : Mixed : 3D	0.011	0.00	35480	3.70	<0.001
	PD : Diffuse : 3D	0.001	0.00	35390	0.41	0.682

Edge Disruption (L GabRat)*

Table S2.7. Stats output for the effect edge disruption, lighting and background 3D variation on fitness, excluding outliers. Linear mixed model formula:

lmer(log(SurvivalTime) ~ GabRat * Light * 3D$_{Variation}$ + (1|Habitat) + (1|Population) …

	Predictor	β	SE	DF	t	p value
log Fitness (Capture Time)	Intercept	0.469	0.02	33	28.08	<0.001
	L GabRat	0.040	0.00	33860	19.93	<0.001
	Mixed	-0.021	0.01	41	-2.12	0.041
	Diffuse	-0.016	0.01	42	-1.57	0.125
	Mean 3D Var	0.029	0.02	32	1.67	0.104
	Gab : Mixed	-0.009	0.00	31400	-3.32	0.001
	Gab : Diffuse	-0.014	0.00	34020	-5.17	<0.001
	Gab : 3D	0.011	0.00	36190	5.09	<0.001
	Mixed : 3D	-0.021	0.01	41	-2.12	0.040
	Diffuse : 3D	-0.025	0.01	41	-2.46	0.018
	Gab : Mixed : 3D	-0.002	0.00	35750	-0.83	0.406
	Gab : Diffuse : 3D	-0.003	0.00	36110	-1.16	0.245

A4.2.4 Phenotype Metrics

PC Contributions

Table S2.8. Contributions to principal components (PC), PC1 and PC2 shown in ranked absolute order for variables that contribute greater than equal to 0.10. Positive values are shown in blue, negative values in red. Negative verticalness indicates increasing horizontalness.

Variable	PC1 Contribution
Contrast 1/2 b*	0.206
Verticalness 1/4 b*	-0.205
Verticalness 1/2 b*	-0.200
Contrast 1/4 b*	0.197
Verticalness 1/8 b*	-0.195
Contrast 1/8 b*	0.193
Contrast 1/2 L*	0.193
Contrast 1/1 b*	0.193
Verticalness 1/4 L*	-0.191
Verticalness 1/1 b*	-0.191
Verticalness 1/2 L*	-0.191
Contrast 1/4 L*	0.185
Contrast 1/2 a*	0.181
Verticalness 1/4 a*	-0.181
Contrast 1/1 L*	0.180
Verticalness 1/8 a*	-0.179
Verticalness 1/2 a*	-0.179
Verticalness 1/1 L*	-0.177
Contrast 1/8 a*	0.176
Verticalness 1/8 L*	-0.175
Contrast 1/1 a*	0.175
Verticalness 1/1 a*	-0.174
Contrast 1/4 a*	0.172
Contrast 1/8 L*	0.162
Contrast 1/16 b*	0.152
Contrast 1/16 a*	0.143
Contrast 1/16 L*	0.126
Contrast 1/32 b*	0.117
Contrast 1/32 a*	0.113
Contrast 1/32 L*	0.109

Variable	PC2 Contribution
Contrast 1/16 L*	0.203
Contrast 1/16 b*	0.199
Verticalness 1/8 a*	0.194
Verticalness 1/16 b*	0.192
Contrast 1/8 L*	0.190
Verticalness 1/8 b*	0.185
Contrast 1/8 b*	0.179
Verticalness 1/16 L*	0.176
Verticalness 1/16 a*	0.174
Verticalness 1/4 a*	0.172
Contrast 1/32 L*	0.172
Verticalness 1/2 a*	0.169
Contrast 1/32 b*	0.168
Verticalness 1/8 L*	0.167
Contrast 1/16 a*	0.164
Verticalness 1/4 b*	0.161
Contrast 1/4 L*	0.160
Verticalness 1/2 b*	0.159
Verticalness 1/1 a*	0.157
Verticalness 1/4 L*	0.157
Verticalness 1/32 b*	0.155
Verticalness 1/1 b*	0.153
Verticalness 1/2 L*	0.153
Verticalness 1/32 L*	0.146
Contrast 1/4 b*	0.144
Contrast 1/8 a*	0.142
Contrast 1/2 L*	0.142
Directionality 1/32 L*	0.138
Directionality 1/32 b*	0.137
Contrast 1/32 a*	0.136
Verticalness 1/1 L*	0.136
Verticalness 1/32 a*	0.129
Contrast 1/2 b*	0.127
Directionality 1/32 a*	0.120
Contrast 1/1 L*	0.117
Contrast 1/4 a*	0.111
Contrast 1/1 b*	0.109
Contrast 1/2 a*	0.100

Table S2.9. Contributions to principal components (PC), PC3 and PC4 shown in ranked absolute order for variables that contribute greater than equal to 0.10. Positive values are shown in blue, negative values in red. Negative verticalness indicates increasing horizontalness.

Variable	PC3 Contribution
Contrast 1/2 a*	-0.361
Contrast 1/4 a*	-0.356
Contrast 1/8 a*	-0.340
Contrast 1/1 a*	-0.313
Contrast 1/16 a*	-0.297
Contrast 1/32 a*	-0.297
Contrast 1/16 b*	0.137
Verticalness 1/2 a*	0.130
Contrast 1/32 b*	0.130
Contrast 1/1 L*	0.127
Contrast 1/8 b*	0.126
Contrast 1/2 L*	0.126
Verticalness 1/4 a*	0.125
Verticalness 1/1 a*	0.125
Contrast 1/2 b*	0.122
Contrast 1/1 b*	0.120
Contrast 1/8 L*	0.116
Contrast 1/16 L*	0.116
Verticalness 1/1 L*	-0.111
Contrast 1/32 L*	0.110
Contrast 1/4 b*	0.108
Verticalness 1/2 L*	-0.108
Contrast 1/4 L*	0.106
Verticalness 1/8 a*	0.104

Variable	PC4 Contribution
Directionality 1/32 a*	0.297
Directionality 1/32 b*	0.285
Directionality 1/32 L*	0.277
Verticalness 1/32 L*	0.275
Verticalness 1/32 b*	0.265
Verticalness 1/32 a*	0.257
Directionality 1/16 a*	0.235
Directionality 1/16 b*	0.222
Directionality 1/16 L*	0.214
Verticalness 1/16 L*	0.196
Verticalness 1/16 b*	0.195
Verticalness 1/2 a*	-0.155
Verticalness 1/16 a*	0.152
Verticalness 1/4 a*	-0.149
Contrast 1/4 b*	-0.142
Verticalness 1/1 a*	-0.139
Mean L*	-0.135
Contrast 1/2 b*	-0.133
Directionality 1/8 a*	0.132
Directionality 1/8 b*	0.112
Contrast 1/8 b*	-0.111
Contrast 1/1 b*	-0.111
Contrast 1/4 L*	-0.101

PC1 Model

Table S2.10. Stats output for the effect number of generations, lighting and background 3D variation on the targets PC1 value, excluding outliers. Linear mixed model formula:

lmer(PC1 ~ Generation * Light * $3D_{Variation}$ + (1|Habitat) + (1|Population) …

	Predictor	β	SE	DF	t	p value
PC1	Intercept	0.108	0.06	66	1.90	0.062
	Generation	-0.042	0.01	36210	-4.97	<0.001
	Mixed	-0.059	0.08	66	-0.73	0.465
	Diffuse	-0.276	0.08	66	-3.37	0.001
	Mean 3D Var	-0.054	0.06	66	-0.94	0.351
	Gen : Mixed	-0.096	0.01	36210	-8.04	<0.001

	Gen : Diffuse	-0.201	0.01	36210	-16.63	<0.001
	Gen : 3D	0.026	0.01	36210	3.04	0.002
	Mixed : 3D	0.123	0.08	66	1.51	0.135
	Diffuse : 3D	-0.009	0.08	66	-0.12	0.908
	Gen : Mixed : 3D	0.015	0.01	36210	1.24	0.214
	Gen : Diffuse : 3D	-0.072	0.01	36210	-5.99	<0.001

A4.2.5 Countershading

Table S2.11. Stats output for the effect number of generations, lighting and background 3D variation on the targets shift in luminance gradient from the grey target to the rendered target, excluding outliers. Linear mixed model formula: lmer(CS_Gradient ~ Generation * Light * $3D_{Variation}$ + (1|Habitat) + (1|Population) …

	Predictor	β	SE	DF	t	p value
Gradient Shift	Intercept	-0.124	0.12	31	-1.00	0.323
	Generation	0.022	0.00	36210	20.99	<0.001
	Mixed	-0.034	0.06	46	-0.57	0.573
	Diffuse	-0.053	0.06	46	-0.84	0.405
	Mean 3D Var	0.270	0.13	30	2.09	0.045
	Gen : Mixed	-0.008	0.00	36210	-5.36	<0.001
	Gen : Diffuse	-0.018	0.00	36210	-12.00	<0.001
	Gen : 3D	0.011	0.00	36210	10.11	<0.001
	Mixed : 3D	-0.135	0.06	46	-2.25	0.029
	Diffuse : 3D	-0.037	0.06	46	-0.60	0.550
	Gen : Mixed : 3D	0.000	0.00	36210	0.28	0.777
	Gen : Diffuse : 3D	-0.008	0.00	36210	-5.18	<0.001

A4.2.6 Pattern Direction

Directionality

Table S2.12. Stats output for the effect number of generations, lighting and background 3D variation on the directionality of the targets pattern at 1/32x, excluding outliers. Linear mixed model formula: lmer(CS_Gradient ~ Generation * Light * $3D_{Variation}$ + (1|Habitat) + (1|Population) …

	Predictor	β	SE	DF	t	p value
Directionality 1/32	Intercept	0.114	0.06	66	1.84	0.070
	Generation	0.023	0.01	36210	2.81	0.005
	Mixed	-0.054	0.09	66	-0.61	0.543
	Diffuse	-0.299	0.09	66	-3.34	0.001
	Mean 3D Var	0.166	0.06	66	2.67	0.010
	Gen : Mixed	-0.004	0.01	36210	-0.30	0.762
	Gen : Diffuse	-0.126	0.01	36210	-10.46	<0.001
	Gen : 3D	0.065	0.01	36210	7.80	<0.001
	Mixed : 3D	-0.233	0.09	66	-2.63	0.011
	Diffuse : 3D	-0.175	0.09	66	-1.96	0.055
	Gen : Mixed : 3D	-0.095	0.01	36210	-7.93	<0.001
	Gen : Diffuse : 3D	-0.096	0.01	36210	-7.95	<0.001

Verticality

Table S2.13. Stats output for the effect number of generations, lighting and background 3D variation on the verticality of the targets pattern at 1/32x, excluding outliers. Linear mixed model formula: lmer(CS_Gradient ~ Generation * Light * $3D_{Variation}$ + (1|Habitat) + (1|Population) …

	Predictor	β	SE	DF	t	p value
Directionality 1/32	Intercept	0.073	0.05	66	1.42	0.162
	Generation	0.086	0.01	36210	10.09	<0.001
	Mixed	-0.087	0.07	45	-1.20	0.237
	Diffuse	-0.138	0.07	49	-1.89	0.065
	Mean 3D Var	0.158	0.05	66	3.02	0.004
	Gen : Mixed	-0.031	0.01	36210	-2.58	0.010
	Gen : Diffuse	-0.073	0.01	36210	-5.94	<0.001
	Gen : 3D	0.095	0.01	36210	11.10	<0.001
	Mixed : 3D	-0.280	0.07	44	-3.84	<0.001
	Diffuse : 3D	-0.182	0.07	45	-2.49	0.017
	Gen : Mixed : 3D	-0.176	0.01	36210	-14.35	<0.001
	Gen : Diffuse : 3D	-0.126	0.01	36210	-10.26	<0.001

Edge Enhancement

Table S2.14. Stats output for the effect number of generations, lighting and background 3D variation on the edge enhancement of the targets (change in luminance contrast at 1/64x scale), excluding outliers. Linear mixed model formula: lmer(CS_Gradient ~ Generation * Light * $3D_{Variation}$ + (1|Habitat) + (1|Population) …

	Predictor	β	SE	DF	t	p value
Edge Enhancement	Intercept	0.040	0.08	60	0.49	0.628
	Generation	0.043	0.01	36210	5.31	<0.001
	Mixed	-0.014	0.10	40	-0.14	0.887
	Diffuse	-0.091	0.10	43	-0.88	0.383
	Mean 3D Var	0.109	0.08	58	1.32	0.193
	Gen : Mixed	0.024	0.01	36210	2.09	0.037
	Gen : Diffuse	-0.030	0.01	36210	-2.54	0.011
	Gen : 3D	0.027	0.01	36210	3.28	0.001
	Mixed : 3D	0.050	0.10	39	0.50	0.622
	Diffuse : 3D	-0.120	0.10	40	-1.18	0.247
	Gen : Mixed : 3D	0.077	0.01	36210	6.59	<0.001
	Gen : Diffuse : 3D	-0.014	0.01	36210	-1.21	0.228
