# Supplementary figures and images for: Shining a light on camouflage evolution: Using genetic algorithms to determine the effects of geometry and lighting on optimal camouflage
Source: PLoS One. 2026 Apr 29;21(4):e0346231. doi: 10.1371/journal.pone.0346231 (PMC13127904; doi:10.1371/journal.pone.0346231)

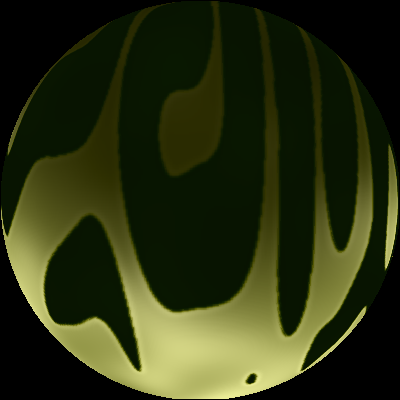

Supplement: S3 File — Zip containing all of the data frames and R code necessary to perform our statistical analyses. (ZIP) [file pone.0346231.s003.zip › S3_File/Sample_Evolved_Patterns/01_grassWildFlowers/1_Natural/Gen20_Mut0_ID4.tif]

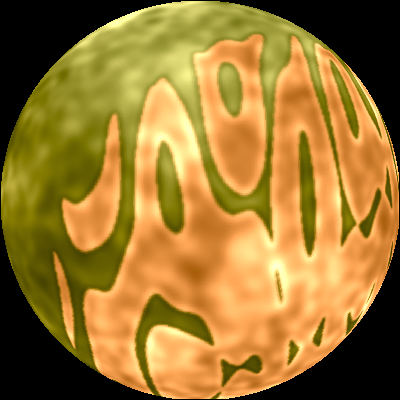

Supplement: S3 File — Zip containing all of the data frames and R code necessary to perform our statistical analyses. (ZIP) [file pone.0346231.s003.zip › S3_File/Sample_Evolved_Patterns/01_grassWildFlowers/2_Diffuse/Gen20_Mut0_ID0.tif]

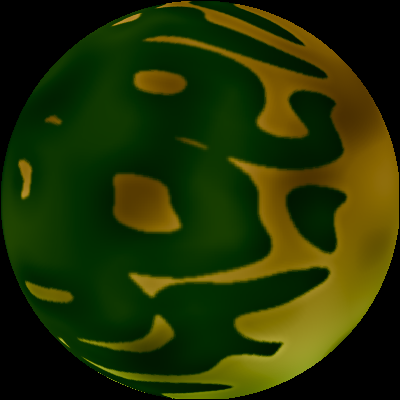

Supplement: S3 File — Zip containing all of the data frames and R code necessary to perform our statistical analyses. (ZIP) [file pone.0346231.s003.zip › S3_File/Sample_Evolved_Patterns/01_grassWildFlowers/3_Hybrid/Gen20_Mut0_ID3.tif]

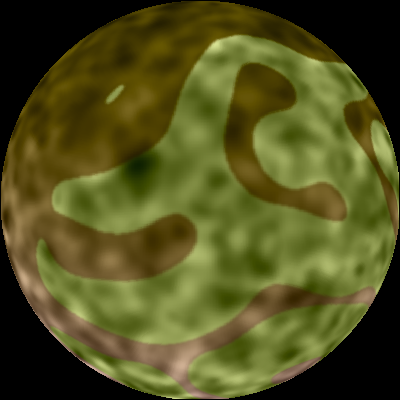

Supplement: S3 File — Zip containing all of the data frames and R code necessary to perform our statistical analyses. (ZIP) [file pone.0346231.s003.zip › S3_File/Sample_Evolved_Patterns/02_grassMowShort/1_Natural/Gen20_Mut0_ID4.tif]

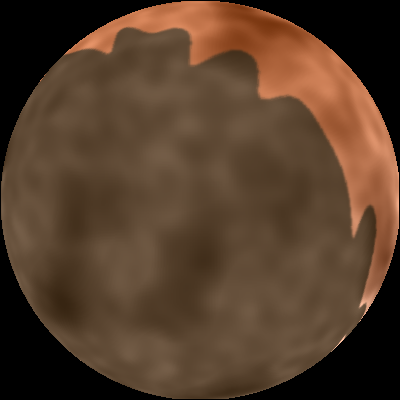

Supplement: S3 File — Zip containing all of the data frames and R code necessary to perform our statistical analyses. (ZIP) [file pone.0346231.s003.zip › S3_File/Sample_Evolved_Patterns/02_grassMowShort/2_Diffuse/Gen18_Mut0_ID1.tif]

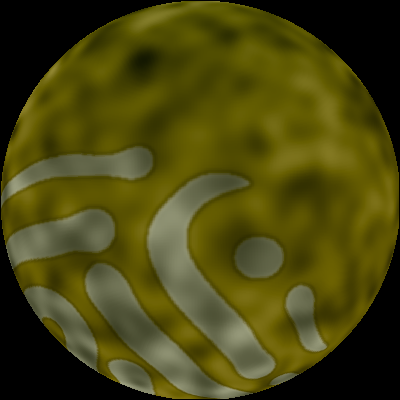

Supplement: S3 File — Zip containing all of the data frames and R code necessary to perform our statistical analyses. (ZIP) [file pone.0346231.s003.zip › S3_File/Sample_Evolved_Patterns/02_grassMowShort/3_Hybrid/Gen19_Mut0_ID1.tif]

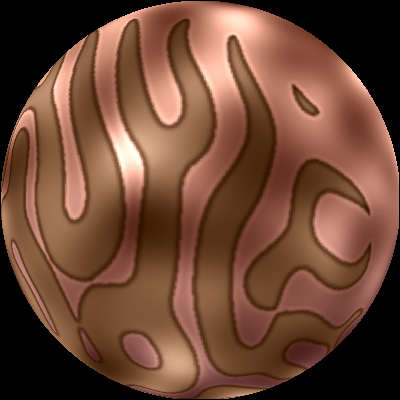

Supplement: S3 File — Zip containing all of the data frames and R code necessary to perform our statistical analyses. (ZIP) [file pone.0346231.s003.zip › S3_File/Sample_Evolved_Patterns/03_woodLeaflitter/1_Natural/Gen20_Mut0_ID11.tif]

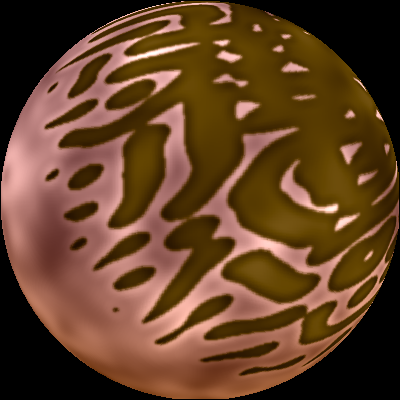

Supplement: S3 File — Zip containing all of the data frames and R code necessary to perform our statistical analyses. (ZIP) [file pone.0346231.s003.zip › S3_File/Sample_Evolved_Patterns/03_woodLeaflitter/2_Diffuse/Gen18_Mut0_ID1.tif]

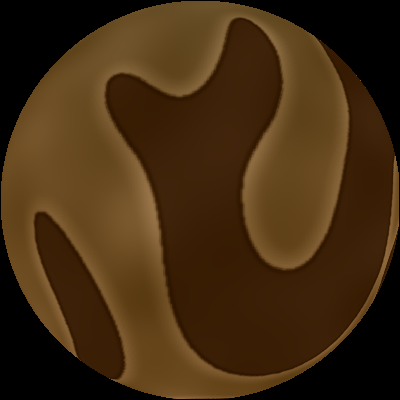

Supplement: S3 File — Zip containing all of the data frames and R code necessary to perform our statistical analyses. (ZIP) [file pone.0346231.s003.zip › S3_File/Sample_Evolved_Patterns/03_woodLeaflitter/3_Hybrid/Gen19_Mut0_ID4.tif]

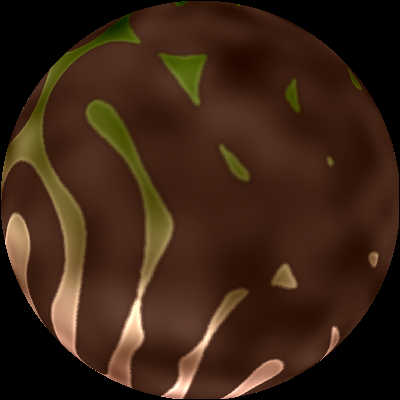

Supplement: S3 File — Zip containing all of the data frames and R code necessary to perform our statistical analyses. (ZIP) [file pone.0346231.s003.zip › S3_File/Sample_Evolved_Patterns/04_woodTrail/1_Natural/Gen18_Mut0_ID0.tif]

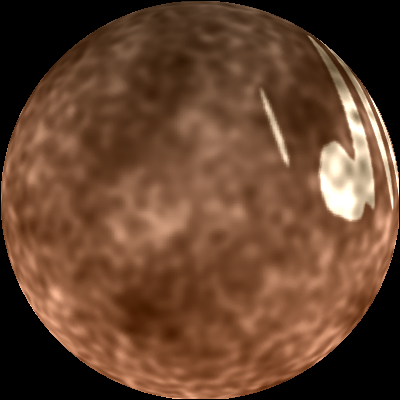

Supplement: S3 File — Zip containing all of the data frames and R code necessary to perform our statistical analyses. (ZIP) [file pone.0346231.s003.zip › S3_File/Sample_Evolved_Patterns/04_woodTrail/2_Diffuse/Gen20_Mut0_ID6.tif]

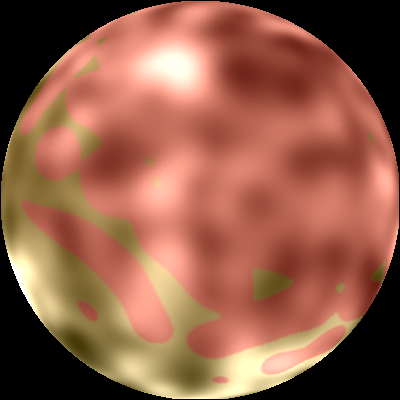

Supplement: S3 File — Zip containing all of the data frames and R code necessary to perform our statistical analyses. (ZIP) [file pone.0346231.s003.zip › S3_File/Sample_Evolved_Patterns/04_woodTrail/3_Hybrid/Gen20_Mut0_ID11.tif]

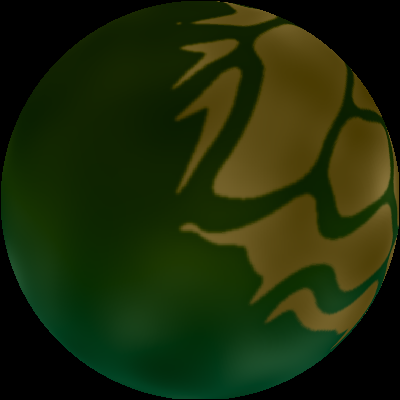

Supplement: S3 File — Zip containing all of the data frames and R code necessary to perform our statistical analyses. (ZIP) [file pone.0346231.s003.zip › S3_File/Sample_Evolved_Patterns/05_grassTrampled/1_Natural/Gen20_Mut0_IDR1.tif]

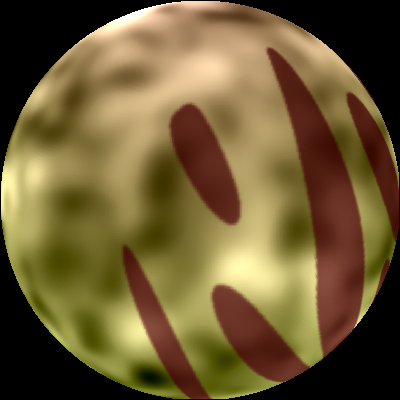

Supplement: S3 File — Zip containing all of the data frames and R code necessary to perform our statistical analyses. (ZIP) [file pone.0346231.s003.zip › S3_File/Sample_Evolved_Patterns/05_grassTrampled/2_Diffuse/Gen19_Mut0_ID9.tif]

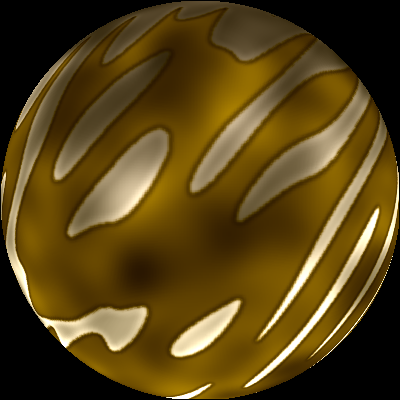

Supplement: S3 File — Zip containing all of the data frames and R code necessary to perform our statistical analyses. (ZIP) [file pone.0346231.s003.zip › S3_File/Sample_Evolved_Patterns/05_grassTrampled/3_Hybrid/Gen20_Mut0_ID7.tif]

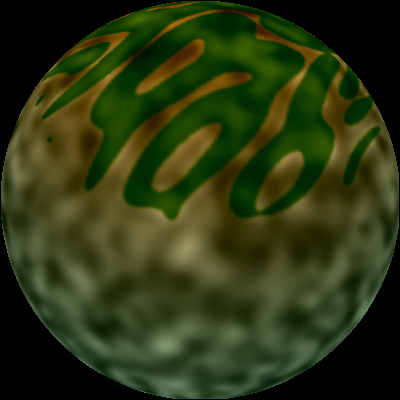

Supplement: S3 File — Zip containing all of the data frames and R code necessary to perform our statistical analyses. (ZIP) [file pone.0346231.s003.zip › S3_File/Sample_Evolved_Patterns/06_grassTopped/1_Natural/Gen19_Mut0_ID4.tif]

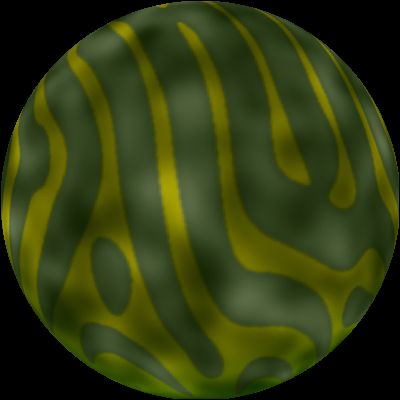

Supplement: S3 File — Zip containing all of the data frames and R code necessary to perform our statistical analyses. (ZIP) [file pone.0346231.s003.zip › S3_File/Sample_Evolved_Patterns/06_grassTopped/2_Diffuse/Gen19_Mut0_ID9.tif]

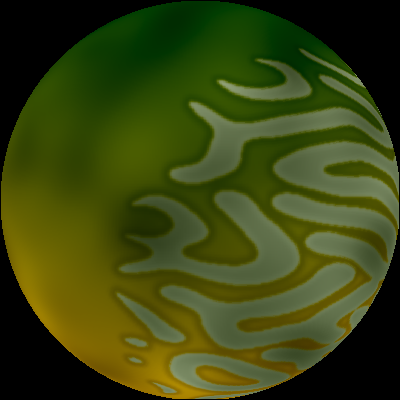

Supplement: S3 File — Zip containing all of the data frames and R code necessary to perform our statistical analyses. (ZIP) [file pone.0346231.s003.zip › S3_File/Sample_Evolved_Patterns/06_grassTopped/3_Hybrid/Gen19_Mut0_ID9.tif]

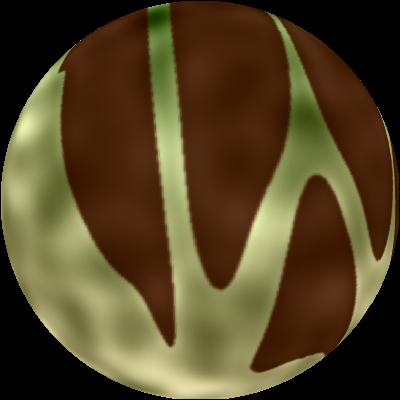

Supplement: S3 File — Zip containing all of the data frames and R code necessary to perform our statistical analyses. (ZIP) [file pone.0346231.s003.zip › S3_File/Sample_Evolved_Patterns/07_woodLichen/1_Natural/Gen20_Mut0_IDR2.tif]

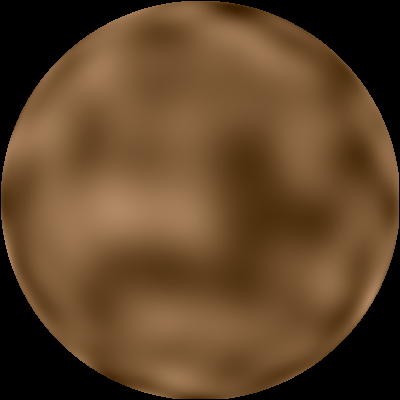

Supplement: S3 File — Zip containing all of the data frames and R code necessary to perform our statistical analyses. (ZIP) [file pone.0346231.s003.zip › S3_File/Sample_Evolved_Patterns/07_woodLichen/2_Diffuse/Gen13_Mut0_ID8.tif]

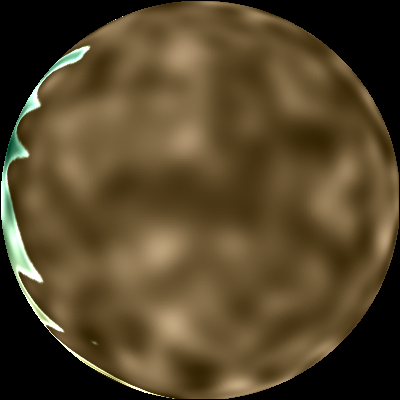

Supplement: S3 File — Zip containing all of the data frames and R code necessary to perform our statistical analyses. (ZIP) [file pone.0346231.s003.zip › S3_File/Sample_Evolved_Patterns/07_woodLichen/3_Hybrid/Gen18_Mut0_ID3.tif]

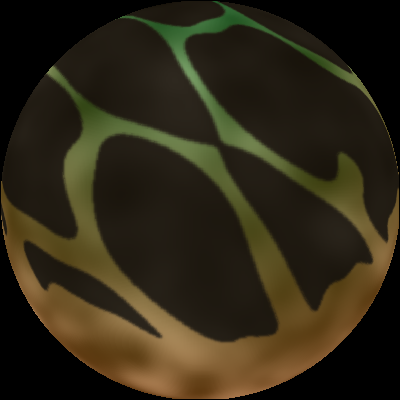

Supplement: S3 File — Zip containing all of the data frames and R code necessary to perform our statistical analyses. (ZIP) [file pone.0346231.s003.zip › S3_File/Sample_Evolved_Patterns/08_grassMowLong/1_Natural/Gen20_Mut0_ID6.tif]

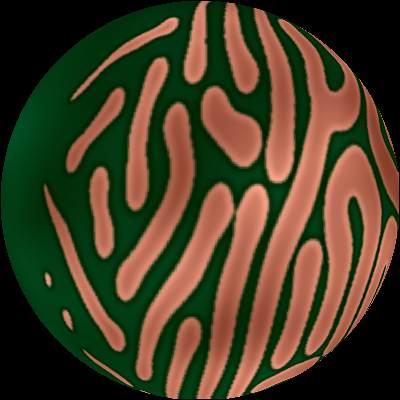

Supplement: S3 File — Zip containing all of the data frames and R code necessary to perform our statistical analyses. (ZIP) [file pone.0346231.s003.zip › S3_File/Sample_Evolved_Patterns/08_grassMowLong/2_Diffuse/Gen19_Mut0_ID9.tif]

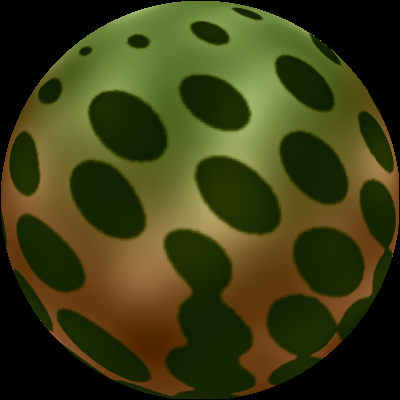

Supplement: S3 File — Zip containing all of the data frames and R code necessary to perform our statistical analyses. (ZIP) [file pone.0346231.s003.zip › S3_File/Sample_Evolved_Patterns/08_grassMowLong/3_Hybrid/Gen20_Mut0_ID1.tif]

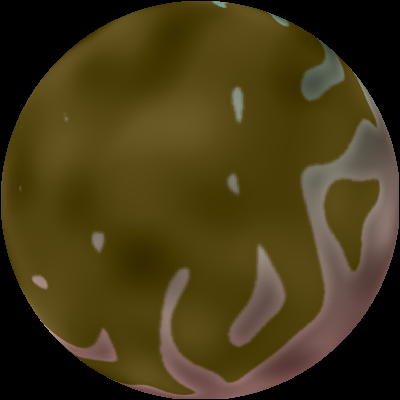

Supplement: S3 File — Zip containing all of the data frames and R code necessary to perform our statistical analyses. (ZIP) [file pone.0346231.s003.zip › S3_File/Sample_Evolved_Patterns/09_woodBramble/1_Natural/Gen20_Mut0_ID9.tif]

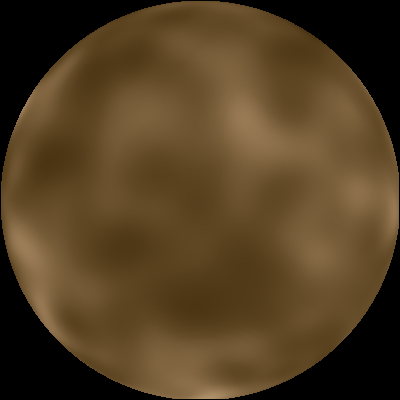

Supplement: S3 File — Zip containing all of the data frames and R code necessary to perform our statistical analyses. (ZIP) [file pone.0346231.s003.zip › S3_File/Sample_Evolved_Patterns/09_woodBramble/2_Diffuse/Gen20_Mut0_ID3.tif]

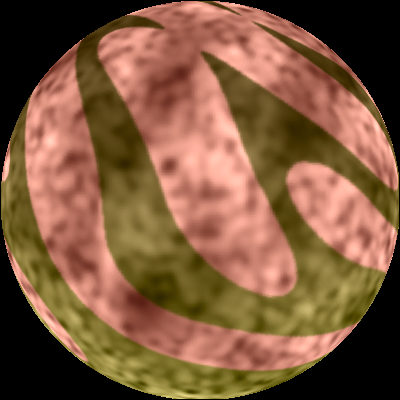

Supplement: S3 File — Zip containing all of the data frames and R code necessary to perform our statistical analyses. (ZIP) [file pone.0346231.s003.zip › S3_File/Sample_Evolved_Patterns/09_woodBramble/3_Hybrid/Gen20_Mut0_ID7.tif]

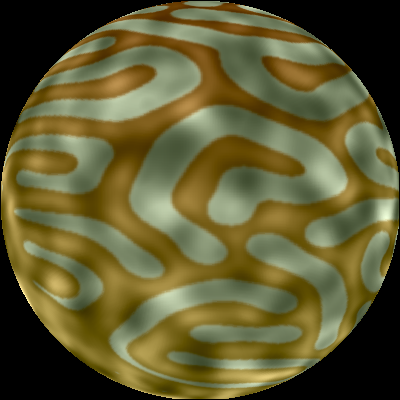

Supplement: S3 File — Zip containing all of the data frames and R code necessary to perform our statistical analyses. (ZIP) [file pone.0346231.s003.zip › S3_File/Sample_Evolved_Patterns/10_woodEdgeGrass/1_Natural/Gen19_Mut0_ID2.tif]

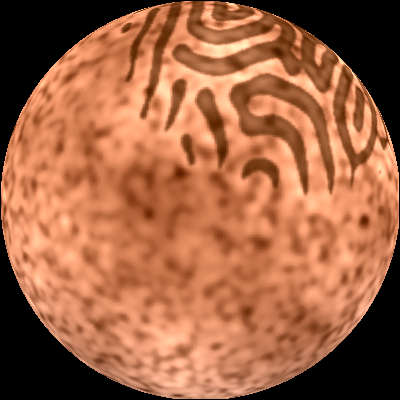

Supplement: S3 File — Zip containing all of the data frames and R code necessary to perform our statistical analyses. (ZIP) [file pone.0346231.s003.zip › S3_File/Sample_Evolved_Patterns/10_woodEdgeGrass/2_Diffuse/Gen20_Mut0_ID10.tif]

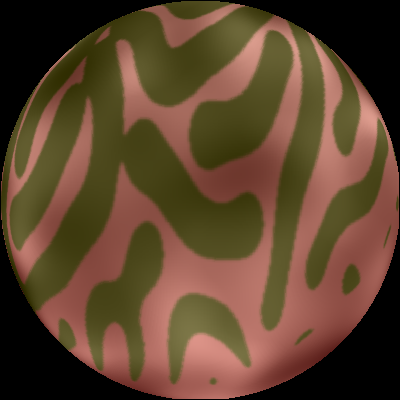

Supplement: S3 File — Zip containing all of the data frames and R code necessary to perform our statistical analyses. (ZIP) [file pone.0346231.s003.zip › S3_File/Sample_Evolved_Patterns/10_woodEdgeGrass/3_Hybrid/Gen20_Mut0_IDR3.tif]

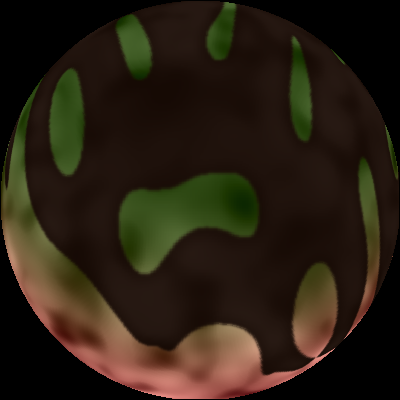

Supplement: S3 File — Zip containing all of the data frames and R code necessary to perform our statistical analyses. (ZIP) [file pone.0346231.s003.zip › S3_File/Sample_Evolved_Patterns/11_heathHeather/1_Natural/Gen20_Mut0_ID2.tif]

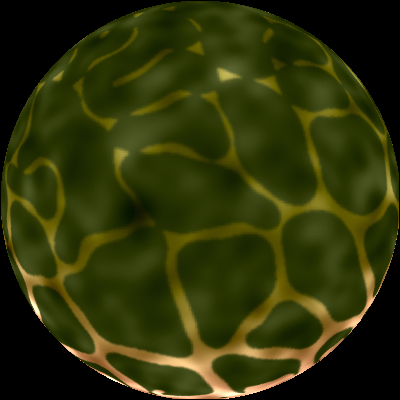

Supplement: S3 File — Zip containing all of the data frames and R code necessary to perform our statistical analyses. (ZIP) [file pone.0346231.s003.zip › S3_File/Sample_Evolved_Patterns/11_heathHeather/2_Diffuse/Gen16_Mut0_ID11.tif]

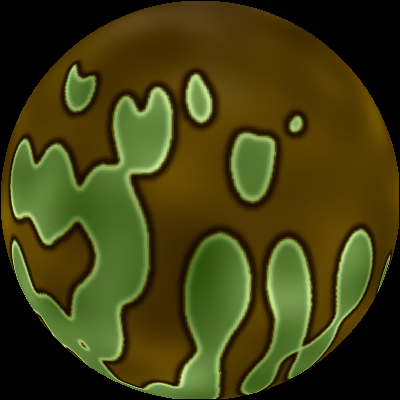

Supplement: S3 File — Zip containing all of the data frames and R code necessary to perform our statistical analyses. (ZIP) [file pone.0346231.s003.zip › S3_File/Sample_Evolved_Patterns/11_heathHeather/3_Hybrid/Gen19_Mut0_ID4.tif]

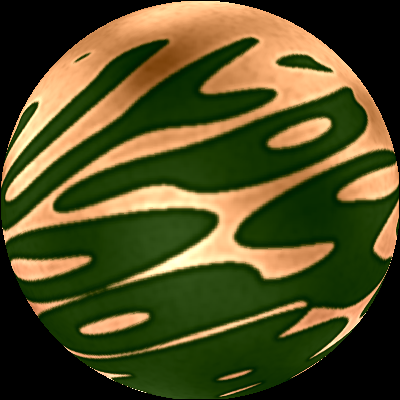

Supplement: S3 File — Zip containing all of the data frames and R code necessary to perform our statistical analyses. (ZIP) [file pone.0346231.s003.zip › S3_File/Sample_Evolved_Patterns/12_heathChalk/1_Natural/Gen16_Mut0_ID6.tif]

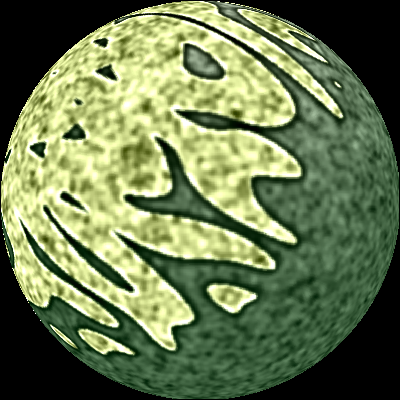

Supplement: S3 File — Zip containing all of the data frames and R code necessary to perform our statistical analyses. (ZIP) [file pone.0346231.s003.zip › S3_File/Sample_Evolved_Patterns/12_heathChalk/2_Diffuse/Gen18_Mut0_ID8.tif]

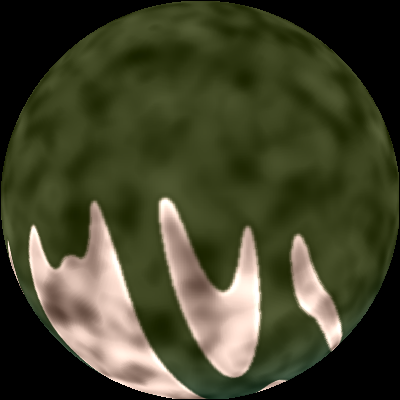

Supplement: S3 File — Zip containing all of the data frames and R code necessary to perform our statistical analyses. (ZIP) [file pone.0346231.s003.zip › S3_File/Sample_Evolved_Patterns/12_heathChalk/3_Hybrid/Gen20_Mut0_ID0.tif]

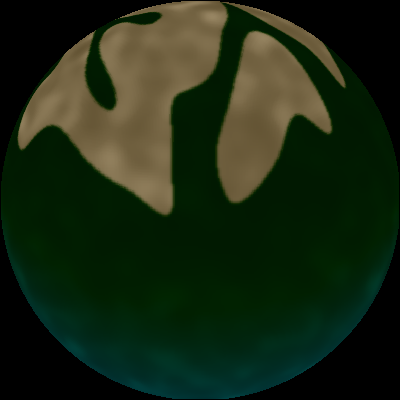

Supplement: S3 File — Zip containing all of the data frames and R code necessary to perform our statistical analyses. (ZIP) [file pone.0346231.s003.zip › S3_File/Sample_Evolved_Patterns/13_heathPond/1_Natural/Gen20_Mut0_ID1.tif]

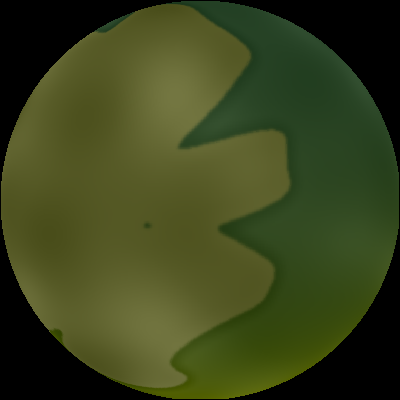

Supplement: S3 File — Zip containing all of the data frames and R code necessary to perform our statistical analyses. (ZIP) [file pone.0346231.s003.zip › S3_File/Sample_Evolved_Patterns/13_heathPond/2_Diffuse/Gen20_Mut0_IDR0.tif]

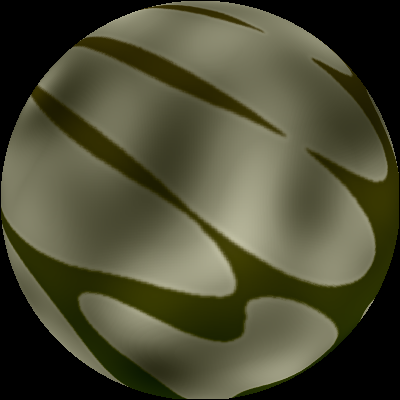

Supplement: S3 File — Zip containing all of the data frames and R code necessary to perform our statistical analyses. (ZIP) [file pone.0346231.s003.zip › S3_File/Sample_Evolved_Patterns/13_heathPond/3_Hybrid/Gen17_Mut0_ID11.tif]

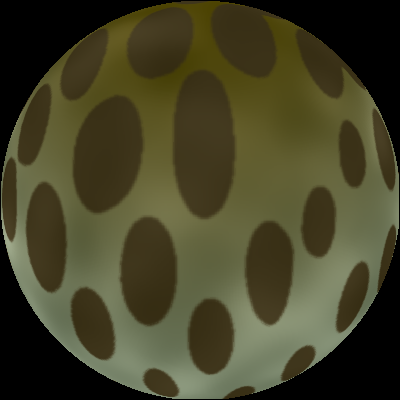

Supplement: S3 File — Zip containing all of the data frames and R code necessary to perform our statistical analyses. (ZIP) [file pone.0346231.s003.zip › S3_File/Sample_Evolved_Patterns/14_heathGrass/1_Natural/Gen19_Mut0_ID0.tif]

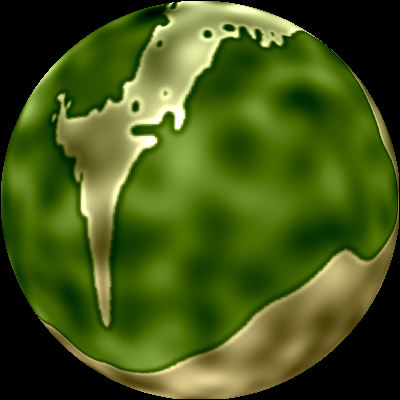

Supplement: S3 File — Zip containing all of the data frames and R code necessary to perform our statistical analyses. (ZIP) [file pone.0346231.s003.zip › S3_File/Sample_Evolved_Patterns/14_heathGrass/2_Diffuse/Gen20_Mut0_ID1.tif]

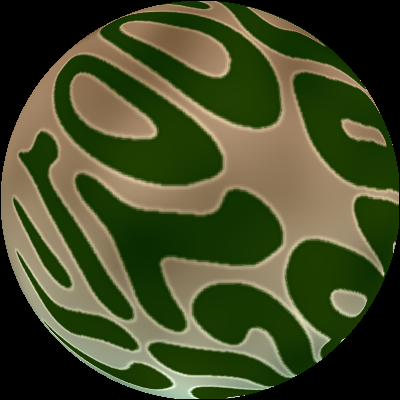

Supplement: S3 File — Zip containing all of the data frames and R code necessary to perform our statistical analyses. (ZIP) [file pone.0346231.s003.zip › S3_File/Sample_Evolved_Patterns/14_heathGrass/3_Hybrid/Gen20_Mut0_ID11.tif]

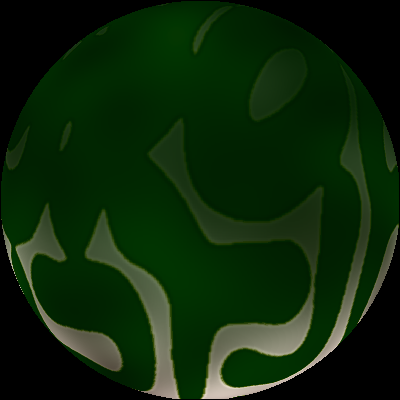

Supplement: S3 File — Zip containing all of the data frames and R code necessary to perform our statistical analyses. (ZIP) [file pone.0346231.s003.zip › S3_File/Sample_Evolved_Patterns/15_heathFerns/1_Natural/Gen19_Mut0_ID9.tif]

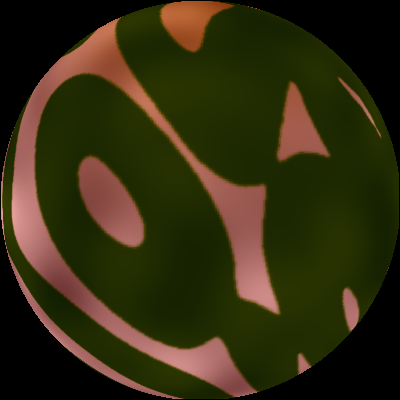

Supplement: S3 File — Zip containing all of the data frames and R code necessary to perform our statistical analyses. (ZIP) [file pone.0346231.s003.zip › S3_File/Sample_Evolved_Patterns/15_heathFerns/2_Diffuse/Gen20_Mut0_ID2.tif]

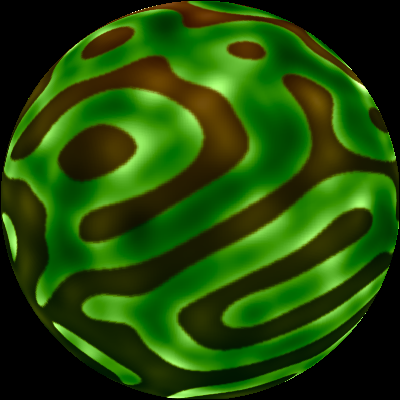

Supplement: S3 File — Zip containing all of the data frames and R code necessary to perform our statistical analyses. (ZIP) [file pone.0346231.s003.zip › S3_File/Sample_Evolved_Patterns/15_heathFerns/3_Hybrid/Gen18_Mut0_ID10.tif]

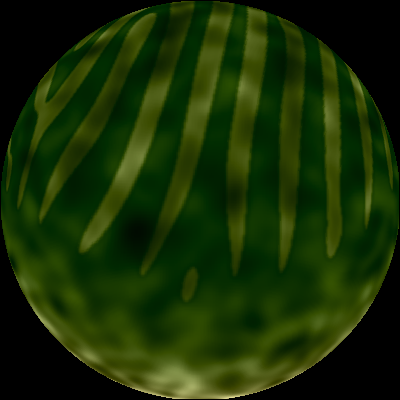

Supplement: S3 File — Zip containing all of the data frames and R code necessary to perform our statistical analyses. (ZIP) [file pone.0346231.s003.zip › S3_File/Sample_Evolved_Patterns/16_heathBog/1_Natural/Gen20_Mut0_ID3.tif]

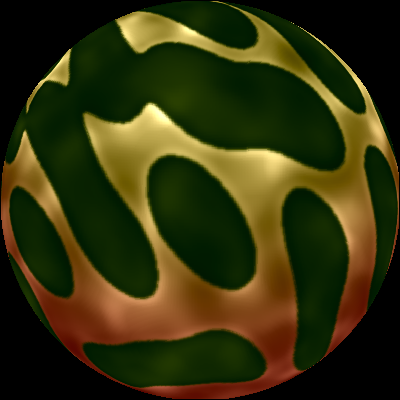

Supplement: S3 File — Zip containing all of the data frames and R code necessary to perform our statistical analyses. (ZIP) [file pone.0346231.s003.zip › S3_File/Sample_Evolved_Patterns/16_heathBog/2_Diffuse/Gen19_Mut0_ID8.tif]

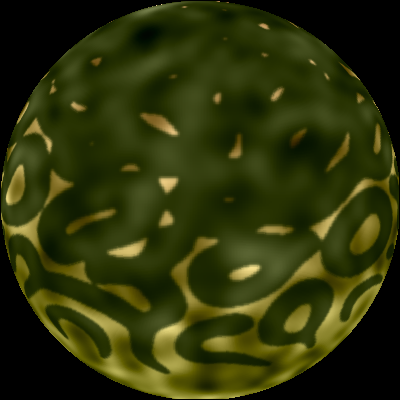

Supplement: S3 File — Zip containing all of the data frames and R code necessary to perform our statistical analyses. (ZIP) [file pone.0346231.s003.zip › S3_File/Sample_Evolved_Patterns/16_heathBog/3_Hybrid/Gen20_Mut0_ID3.tif]

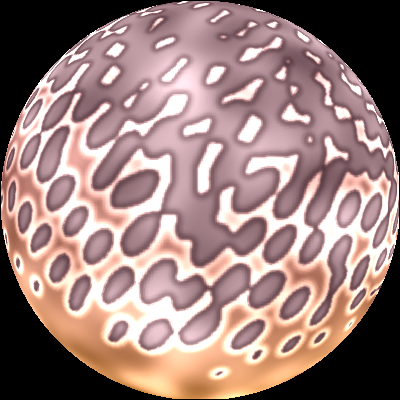

Supplement: S3 File — Zip containing all of the data frames and R code necessary to perform our statistical analyses. (ZIP) [file pone.0346231.s003.zip › S3_File/Sample_Evolved_Patterns/17_heathErosion/1_Natural/Gen20_Mut0_ID0.tif]

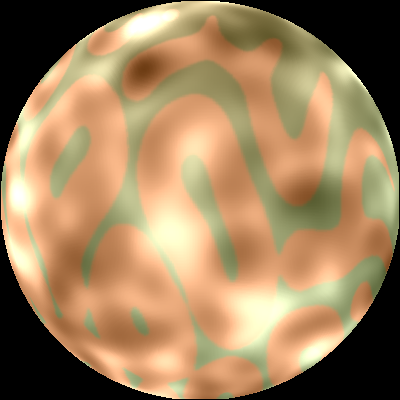

Supplement: S3 File — Zip containing all of the data frames and R code necessary to perform our statistical analyses. (ZIP) [file pone.0346231.s003.zip › S3_File/Sample_Evolved_Patterns/17_heathErosion/2_Diffuse/Gen20_Mut0_ID8.tif]

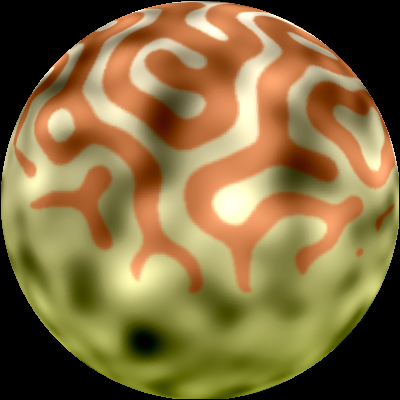

Supplement: S3 File — Zip containing all of the data frames and R code necessary to perform our statistical analyses. (ZIP) [file pone.0346231.s003.zip › S3_File/Sample_Evolved_Patterns/17_heathErosion/3_Hybrid/Gen20_Mut0_ID9.tif]

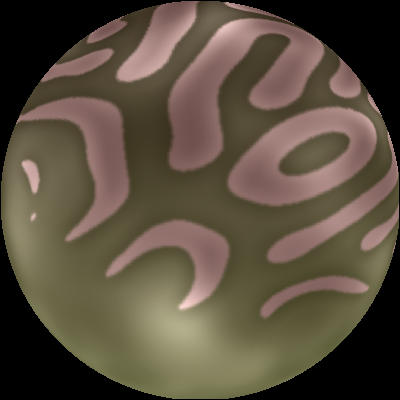

Supplement: S3 File — Zip containing all of the data frames and R code necessary to perform our statistical analyses. (ZIP) [file pone.0346231.s003.zip › S3_File/Sample_Evolved_Patterns/18_heathScrub/1_Natural/Gen20_Mut0_IDR1.tif]

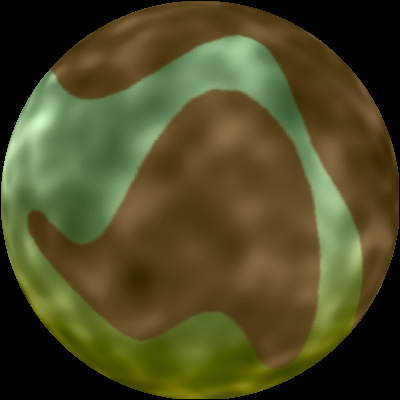

Supplement: S3 File — Zip containing all of the data frames and R code necessary to perform our statistical analyses. (ZIP) [file pone.0346231.s003.zip › S3_File/Sample_Evolved_Patterns/18_heathScrub/2_Diffuse/Gen18_Mut0_ID0.tif]

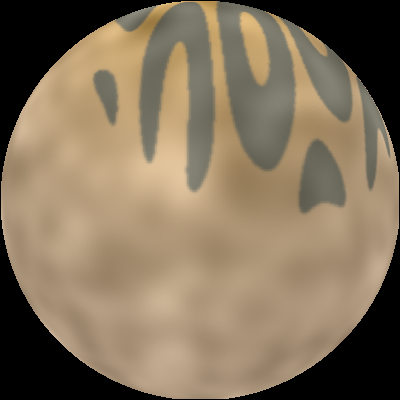

Supplement: S3 File — Zip containing all of the data frames and R code necessary to perform our statistical analyses. (ZIP) [file pone.0346231.s003.zip › S3_File/Sample_Evolved_Patterns/18_heathScrub/3_Hybrid/Gen18_Mut0_ID0.tif]

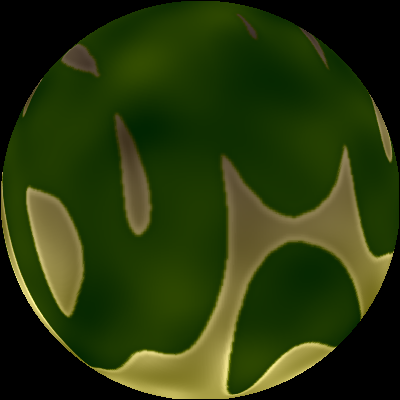

Supplement: S3 File — Zip containing all of the data frames and R code necessary to perform our statistical analyses. (ZIP) [file pone.0346231.s003.zip › S3_File/Sample_Evolved_Patterns/19_woodNettle/1_Natural/Gen17_Mut0_ID6.tif]

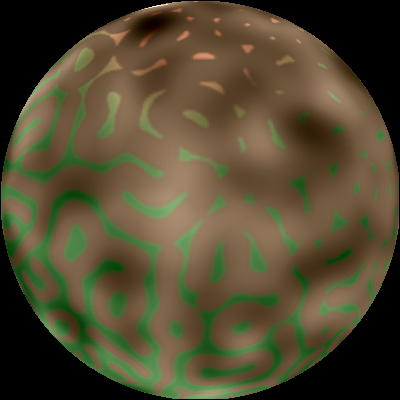

Supplement: S3 File — Zip containing all of the data frames and R code necessary to perform our statistical analyses. (ZIP) [file pone.0346231.s003.zip › S3_File/Sample_Evolved_Patterns/19_woodNettle/2_Diffuse/Gen17_Mut0_ID3.tif]

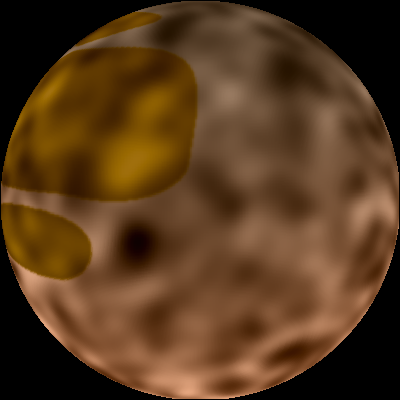

Supplement: S3 File — Zip containing all of the data frames and R code necessary to perform our statistical analyses. (ZIP) [file pone.0346231.s003.zip › S3_File/Sample_Evolved_Patterns/19_woodNettle/3_Hybrid/Gen19_Mut0_ID10.tif]

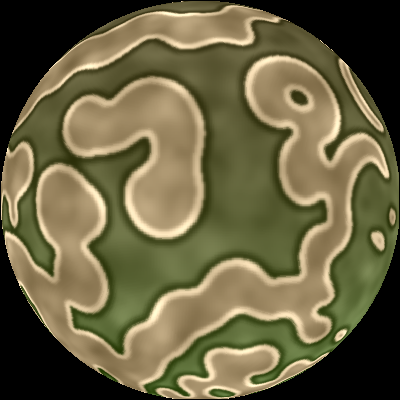

Supplement: S3 File — Zip containing all of the data frames and R code necessary to perform our statistical analyses. (ZIP) [file pone.0346231.s003.zip › S3_File/Sample_Evolved_Patterns/20_estuaryPebble/1_Natural/Gen18_Mut0_ID4.tif]

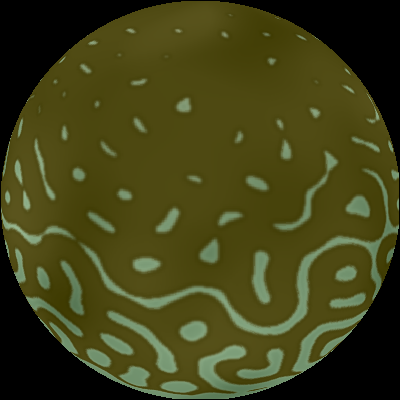

Supplement: S3 File — Zip containing all of the data frames and R code necessary to perform our statistical analyses. (ZIP) [file pone.0346231.s003.zip › S3_File/Sample_Evolved_Patterns/20_estuaryPebble/2_Diffuse/Gen16_Mut0_ID1.tif]

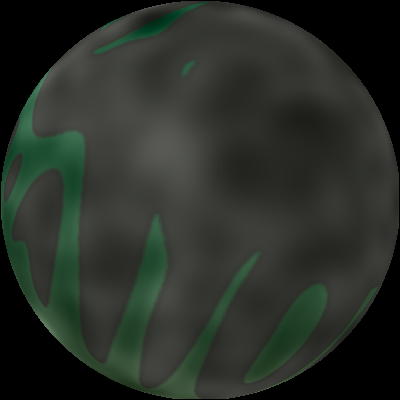

Supplement: S3 File — Zip containing all of the data frames and R code necessary to perform our statistical analyses. (ZIP) [file pone.0346231.s003.zip › S3_File/Sample_Evolved_Patterns/20_estuaryPebble/3_Hybrid/Gen20_Mut0_ID7.tif]

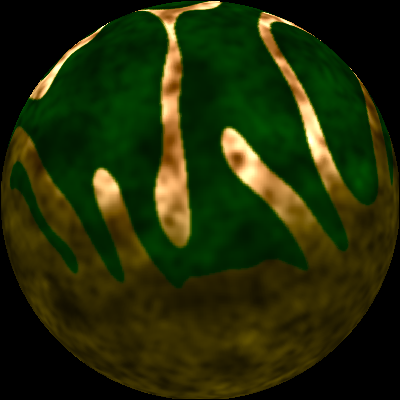

Supplement: S3 File — Zip containing all of the data frames and R code necessary to perform our statistical analyses. (ZIP) [file pone.0346231.s003.zip › S3_File/Sample_Evolved_Patterns/21_estuarySeaweed/1_Natural/Gen20_Mut0_ID10.tif]

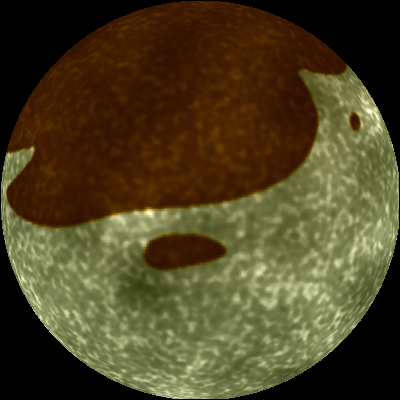

Supplement: S3 File — Zip containing all of the data frames and R code necessary to perform our statistical analyses. (ZIP) [file pone.0346231.s003.zip › S3_File/Sample_Evolved_Patterns/21_estuarySeaweed/2_Diffuse/Gen20_Mut0_ID6.tif]

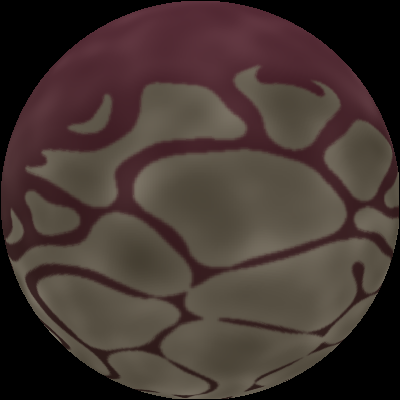

Supplement: S3 File — Zip containing all of the data frames and R code necessary to perform our statistical analyses. (ZIP) [file pone.0346231.s003.zip › S3_File/Sample_Evolved_Patterns/21_estuarySeaweed/3_Hybrid/Gen18_Mut0_ID1.tif]

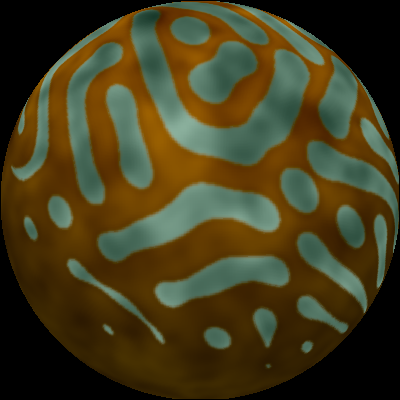

Supplement: S3 File — Zip containing all of the data frames and R code necessary to perform our statistical analyses. (ZIP) [file pone.0346231.s003.zip › S3_File/Sample_Evolved_Patterns/22_estuaryGravel/1_Natural/Gen18_Mut0_ID1.tif]

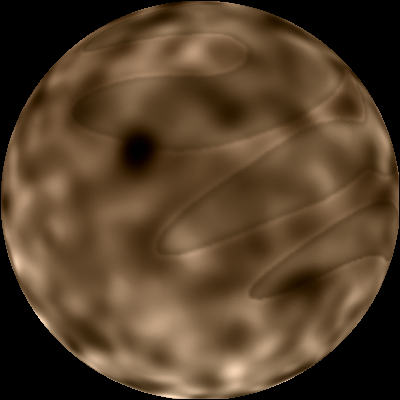

Supplement: S3 File — Zip containing all of the data frames and R code necessary to perform our statistical analyses. (ZIP) [file pone.0346231.s003.zip › S3_File/Sample_Evolved_Patterns/22_estuaryGravel/2_Diffuse/Gen20_Mut0_ID8.tif]

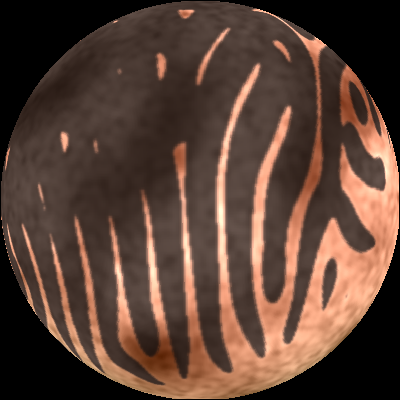

Supplement: S3 File — Zip containing all of the data frames and R code necessary to perform our statistical analyses. (ZIP) [file pone.0346231.s003.zip › S3_File/Sample_Evolved_Patterns/22_estuaryGravel/3_Hybrid/Gen20_Mut0_ID9.tif]

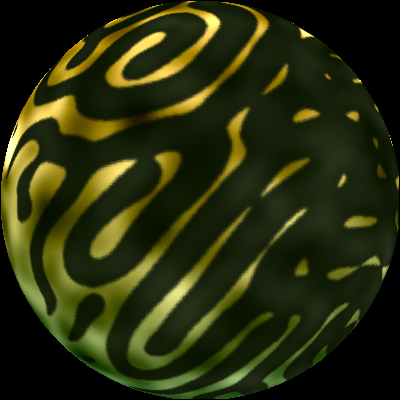

Supplement: S3 File — Zip containing all of the data frames and R code necessary to perform our statistical analyses. (ZIP) [file pone.0346231.s003.zip › S3_File/Sample_Evolved_Patterns/23_estuaryVeg/1_Natural/Gen20_Mut0_ID6.tif]

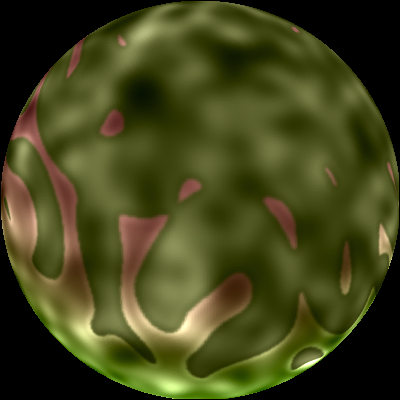

Supplement: S3 File — Zip containing all of the data frames and R code necessary to perform our statistical analyses. (ZIP) [file pone.0346231.s003.zip › S3_File/Sample_Evolved_Patterns/23_estuaryVeg/2_Diffuse/Gen16_Mut0_ID3.tif]

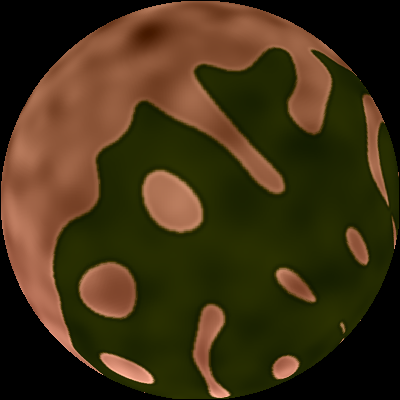

Supplement: S3 File — Zip containing all of the data frames and R code necessary to perform our statistical analyses. (ZIP) [file pone.0346231.s003.zip › S3_File/Sample_Evolved_Patterns/23_estuaryVeg/3_Hybrid/Gen20_Mut0_IDR0.tif]

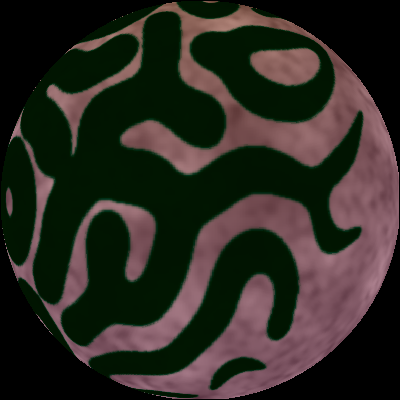

Supplement: S3 File — Zip containing all of the data frames and R code necessary to perform our statistical analyses. (ZIP) [file pone.0346231.s003.zip › S3_File/Sample_Evolved_Patterns/24_estuarySilt/1_Natural/Gen18_Mut0_ID1.tif]

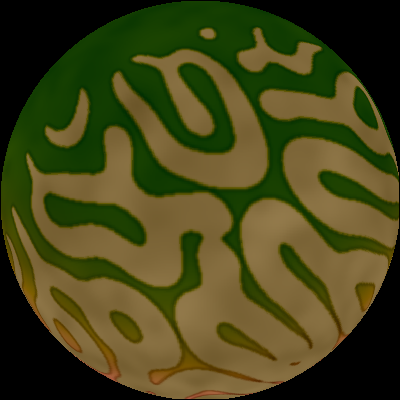

Supplement: S3 File — Zip containing all of the data frames and R code necessary to perform our statistical analyses. (ZIP) [file pone.0346231.s003.zip › S3_File/Sample_Evolved_Patterns/24_estuarySilt/2_Diffuse/Gen19_Mut0_ID9.tif]

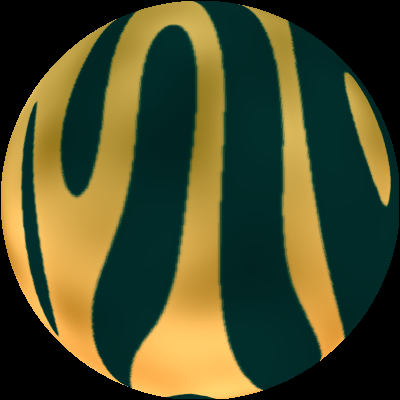

Supplement: S3 File — Zip containing all of the data frames and R code necessary to perform our statistical analyses. (ZIP) [file pone.0346231.s003.zip › S3_File/Sample_Evolved_Patterns/24_estuarySilt/3_Hybrid/Gen20_Mut0_ID9.tif]

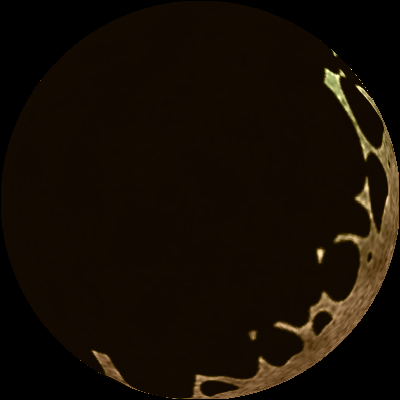

Supplement: S3 File — Zip containing all of the data frames and R code necessary to perform our statistical analyses. (ZIP) [file pone.0346231.s003.zip › S3_File/Sample_Evolved_Patterns/25_farmPlough/1_Natural/Gen20_Mut0_ID1.tif]

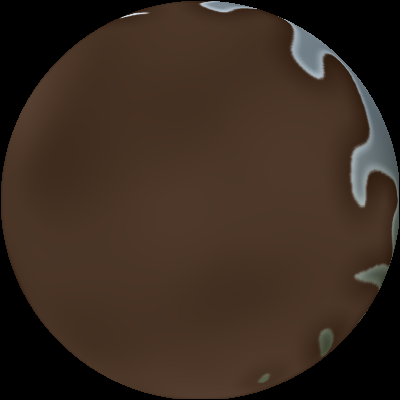

Supplement: S3 File — Zip containing all of the data frames and R code necessary to perform our statistical analyses. (ZIP) [file pone.0346231.s003.zip › S3_File/Sample_Evolved_Patterns/25_farmPlough/2_Diffuse/Gen18_Mut0_IDR1.tif]

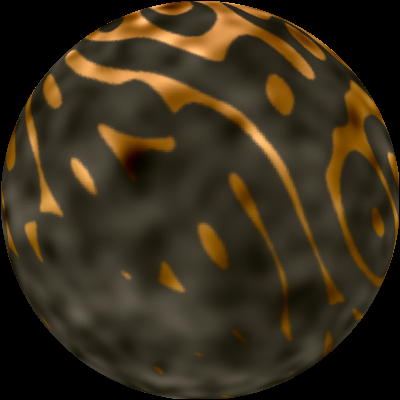

Supplement: S3 File — Zip containing all of the data frames and R code necessary to perform our statistical analyses. (ZIP) [file pone.0346231.s003.zip › S3_File/Sample_Evolved_Patterns/25_farmPlough/3_Hybrid/Gen19_Mut0_ID3.tif]

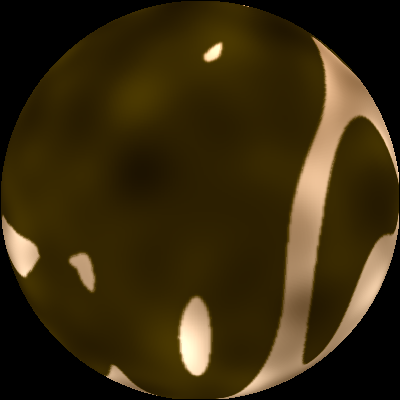

Supplement: S3 File — Zip containing all of the data frames and R code necessary to perform our statistical analyses. (ZIP) [file pone.0346231.s003.zip › S3_File/Sample_Evolved_Patterns/26_woodFlower/1_Natural/Gen19_Mut0_ID2.tif]

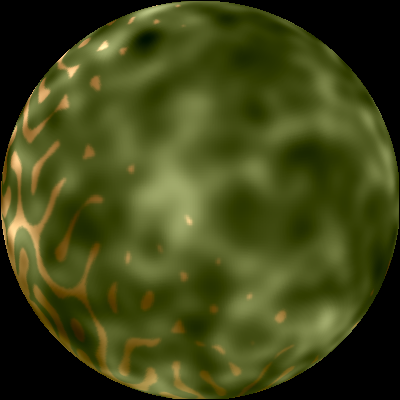

Supplement: S3 File — Zip containing all of the data frames and R code necessary to perform our statistical analyses. (ZIP) [file pone.0346231.s003.zip › S3_File/Sample_Evolved_Patterns/26_woodFlower/2_Diffuse/Gen20_Mut0_ID4.tif]

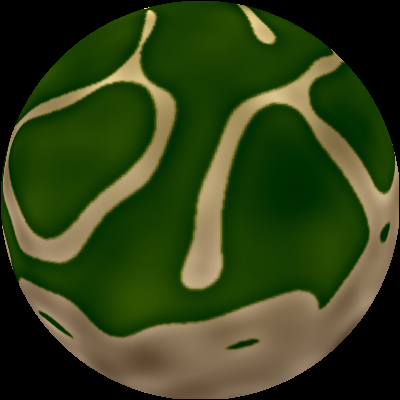

Supplement: S3 File — Zip containing all of the data frames and R code necessary to perform our statistical analyses. (ZIP) [file pone.0346231.s003.zip › S3_File/Sample_Evolved_Patterns/26_woodFlower/3_Hybrid/Gen16_Mut0_IDR1.tif]

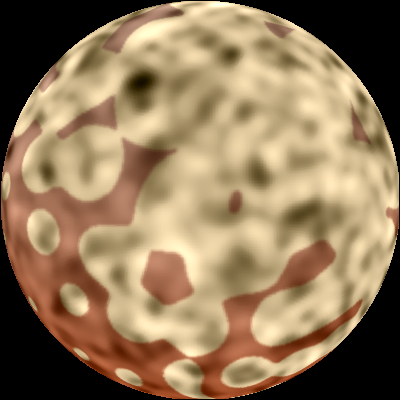

Supplement: S3 File — Zip containing all of the data frames and R code necessary to perform our statistical analyses. (ZIP) [file pone.0346231.s003.zip › S3_File/Sample_Evolved_Patterns/27_gravelMajority/1_Natural/Gen20_Mut0_ID6.tif]

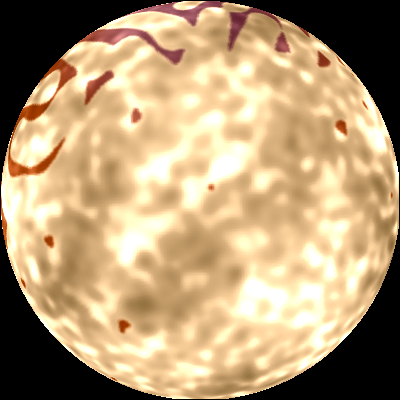

Supplement: S3 File — Zip containing all of the data frames and R code necessary to perform our statistical analyses. (ZIP) [file pone.0346231.s003.zip › S3_File/Sample_Evolved_Patterns/27_gravelMajority/2_Diffuse/Gen20_Mut0_ID10.tif]

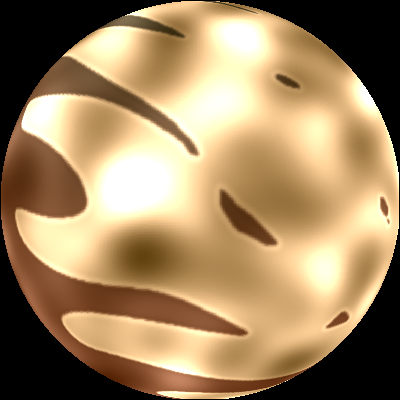

Supplement: S3 File — Zip containing all of the data frames and R code necessary to perform our statistical analyses. (ZIP) [file pone.0346231.s003.zip › S3_File/Sample_Evolved_Patterns/27_gravelMajority/3_Hybrid/Gen16_Mut0_ID6.tif]

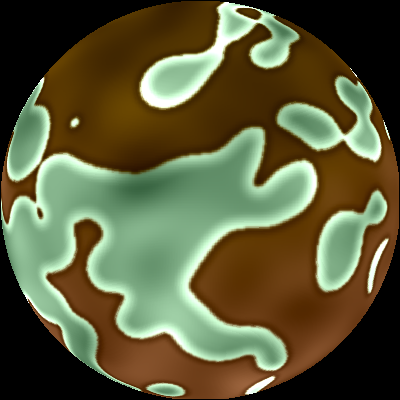

Supplement: S3 File — Zip containing all of the data frames and R code necessary to perform our statistical analyses. (ZIP) [file pone.0346231.s003.zip › S3_File/Sample_Evolved_Patterns/28_gravelMix/1_Natural/Gen18_Mut0_ID11.tif]

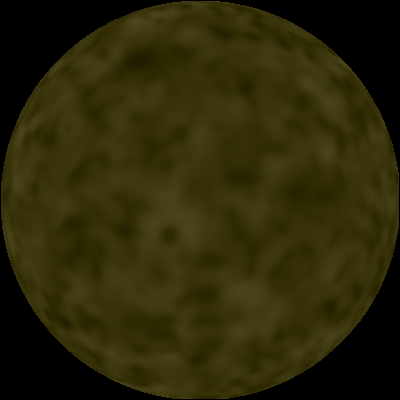

Supplement: S3 File — Zip containing all of the data frames and R code necessary to perform our statistical analyses. (ZIP) [file pone.0346231.s003.zip › S3_File/Sample_Evolved_Patterns/28_gravelMix/2_Diffuse/Gen19_Mut0_IDR0.tif]

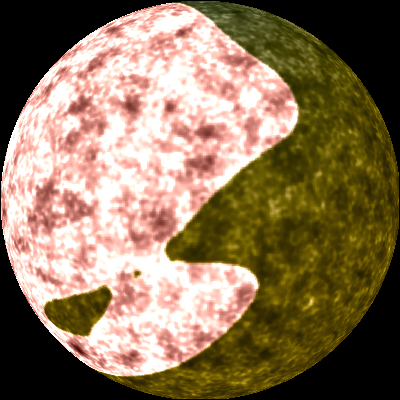

Supplement: S3 File — Zip containing all of the data frames and R code necessary to perform our statistical analyses. (ZIP) [file pone.0346231.s003.zip › S3_File/Sample_Evolved_Patterns/28_gravelMix/3_Hybrid/Gen20_Mut0_ID1.tif]

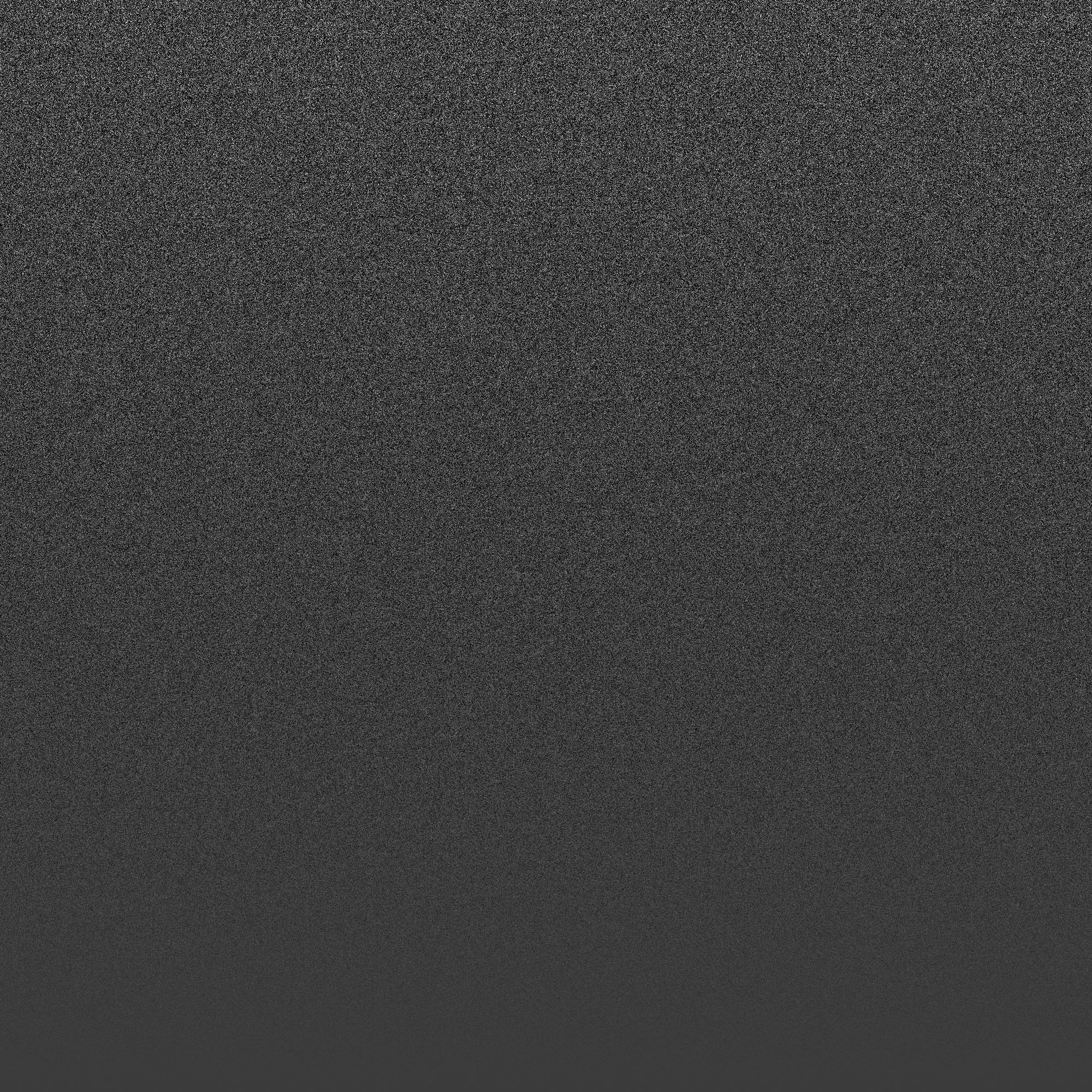

Supplement: S3 File — Zip containing all of the data frames and R code necessary to perform our statistical analyses. (ZIP) [file pone.0346231.s003.zip › S3_File/Supplementary_ImageJ_Plugins/plugins/ButtonGame CamoGen/Patterns/eggPatterns.jpg]

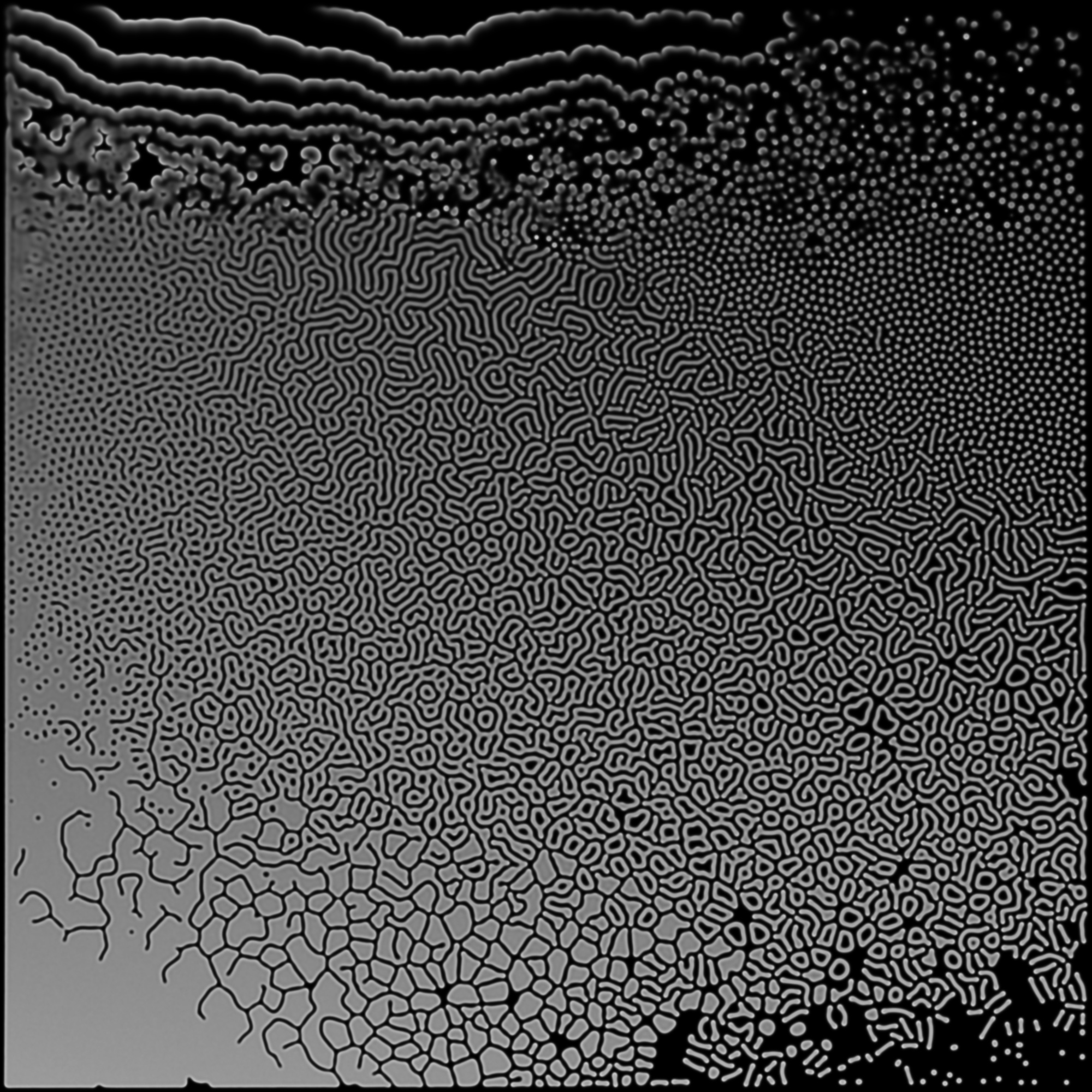

Supplement: S3 File — Zip containing all of the data frames and R code necessary to perform our statistical analyses. (ZIP) [file pone.0346231.s003.zip › S3_File/Supplementary_ImageJ_Plugins/plugins/ButtonGame CamoGen/Patterns/pattern1.jpg]
