# Supplementary material for: Shining a light on camouflage evolution: Using genetic algorithms to determine the effects of geometry and lighting on optimal camouflage
Source: PLoS One. 2026 Apr 29;21(4):e0346231. doi: 10.1371/journal.pone.0346231 (PMC13127904; doi:10.1371/journal.pone.0346231)
Supplement: Attachment — Submitted filename: PONE-D-25-23182_Notes.docx [file pone.0346231.s004.docx]

**PONE-D-25-23182**

The authors present a comprehensive computational approach to assess the efficacy of camouflage strategies in conjunction with changes in light and habitat geometry. Given the numerousness and diversity of interactions between these ecological features in the wild, as well as the difficulty in recreating and standardising these interactions in a non-computational experimental set-up, I agree with the claim by the authors that their approach is the first and most optimal solution for addressing this question. The paper therefore presents a very useful foundation for further camouflage investigation and will be of interest to many readers. I have no major concerns with the content, though I believe the clarity of the writing and punctuation needs to be improved at times (outlined in my minor comments). While I understand that the scale of this study and the nature of the question generates a multitude of different statistical comparisons and outputs, I do also fear that the results section may be slightly overwhelming to some readers. I therefore wonder if these results can be summarised with clearer language (i.e., not using model terms) and infographics, as demonstrated in the predictions table (which was very useful for visualisation).

**Minor comments**

Line 51. Different referencing style for “(Matchette et al., 2018, 2019)”

Line 52. Consider rephrasing to “… maintain effective camouflage, as well as to maximise salience when signalling”

Line 56. Consider rephrasing to “… what should camouflage, which is specialised to either fixed or variable lighting environments, look like”

Line 58. * “On a cloudless day, when the habitat is open, …”

Line 103. * “For example, comparisons of…”

Line 104. There is a big emphasis on countershading within this paper, which is reasonable given its efficacy naturally depends on the light environment. However, the set-up (i.e., viewing targets from a birds-eye-view) is unrepresentative of the most common natural forms of countershading, where the effect is most pronounced when viewed laterally (with the effect representing background matching when viewed from above or below). I agree that, here, a countershading effect may evolve (if direct lighting is present) and that this is informative, but the conditions and context for this emergence here are less naturalistic and I think this needs to be caveated for the reader.

Line 110. * “… enhancement patterns, which pictorially mimic shadows that form at the edges of objects from direct lighting, are harder…”

Line 119. * “…thought to be one factor…”

Line 121. * “change select for background matching…”

Line 126. * “e.g., glossiness (44)”

Line 128. Consider restructuring the sentence introducing the predictions table.

Line 138. I’d move the reference for CamoEvo to directly next to it, then adjust to “which we used to disentangle…”

Line 140. Be consistent with capitalisation for every mention of CamoEvo.

Line 148. * “… by lighting, such as…”

Line 149. This line is a repeat of the earlier statement; consider removing this or the earlier version.

Line 153. * “…directionality, while downwards…”

Line 154. * “… a negative effect; horizontal lines indicate no effect.”

Line 156. The phrase “refer to the axes widely used CIELAB perceptual colour space” needs rewording for increased clarity.

Line 168. Do you have some quantification of how much the intensity of light varied between direct light treatments across habitats?

Line 172. From here on, the use of “Fig” should be adjusted to “Fig.”

Line 174. Did you have some procedure when choosing which habitat patch to photograph (and therefore where the target was placed)?

Line 183. An example would be helpful for “other tall objects”

Line 193. * “glossiness of the background pattern.”

Line 193. Consider rephrasing this sentence e.g., “For full details of this system, see the Supplementary Materials and XXX et al. (XX)”

Line 200. The phrase “were then randomly cropped to create the stimuli” needs more clarity for the reader.

Line 202. Be consistent with language (e.g., “target” or “target button”), or more explicitly introduce the “button”.

Line 208. * “… (DIFFUSE), and then a mixed treatment…”

Line 214. * “.. the previously mentioned stimuli.”

Line 215. What was the source for the existing javascript?

Line 216. * “With this set-up, the goal was to use humans…”

Line 219. * “capturing the targets, one by one, as fast as they could.”

Line 223. * “… displayed in a random order.”

Lines 230-235. I think this section needs a little more clarity. So, if I have this right, ‘response time’ is the time between the start of the trial and the time at which point the cursor breaks the circle, whereas capture time comprises the response time plus the time from circle to the target. It would be good to get a sense of the number of trials that capture time and response time are used respectively. If capture time is used in extreme cases (i.e., delayed or unreasonably fast) as well as for all touch screen interactions (again, an n would be useful), then why not just use capture time throughout (for consistency)? The legend for Figure 1 also states that the selection of targets was based on the time taken to click on the targets, which wouldn’t have been the case for “response time” instances (unless I’ve misunderstood).

Line 234. Be more explicit that the measure is a proxy for survival time (rather than “dubbed”)

Line 237. Wrongly formatted reference.

Line 246. Is “(Gen)” needed here?

Line 302. * “… Dev=1, while lighting treatment was encoded as…”

Lines 302 and beyond. Be consistent with “lighting treatment” – I think it makes it easier to follow.

Line 312. Consider introducing the formula as: “Using lme4 nomenclature, the models were as follows:”. Also, there is no need for the end of this sentence to still be in italics, and can be isolated as a new sentence.

Line 315. * “… geometry (i.e., pattern contrast…”

Line 316. * “… glossiness), we first…”

Line 327. I think the response measures that have been transformed need to be explicitly mentioned (for replicability’s sake)

Line 332. * “… tables are provided in the Supplementary Materials (S2_File).”

Line 336-338. Consider rephrasing this sentence to “To assess whether targets become more camouflaged, we measured the change in survival time (i.e., our fitness proxy) as a function of generation number (Fig. 2).” For the layman (and general readability), I’d also stick to phrases like “generation number” rather than “n-generation” throughout.

Line 337. * “… improved generation number (via a model not using…”

Line 342. Add number of replicates here i.e., “… increase in fitness (n = 3) or decreased in fitness with generation number (n = 12).”

Line 344. Consider rephrasing to “Populations where the generation number lowered survival time were excluded from subsequent analyses, with the exception of the habitat analysis.”

Line 347. Best to choose either variation or complexity (rather than use a forward slash).

Line 360. Sentence ends abruptly after “which”.

Line 365. *than

Line 373. *effect

Line 392-395. Why Ai if only one panel (same applies to later figures)? Bi needs to have slightly more information i.e., including information from the key. The start of the description for Bii is also needs to be made more clear. Change from “pattern match” to “pattern difference” (as this is what is in the figure)

Line 451. * “…for all substrates under both mixed and direct lighting, except gravel under the direct lighting condition”.

Line 453. * high

Line 499. *see

Line 514. * “… of Merilaita’s (3) oft-cited model…”

Line 525. * “… critical relationships between lighting and camouflage with habitat geometry.” (Then delete the rest of that sentence)

Line 545. This paper does not directly test the military aspect, so an additional reference is needed here.

Line 563. * “… and, by extension, make…”

Line 574. * “… has yet…”

Line 577. Is maculation the best word here? This term has been used a few times in the paper, and I think it probably needs to be more clearly defined at its first reference i.e., my image of this word is spots, hence why “directional striped ‘spots’” are hard to visualise

Line 588. * “… turn be aligned with them.”

Line 609. Consider rephrasing to “We predict that simple ecological and behavioural differences…”, mainly because these haven’t been tested.

*Supplementary: S1_File*

Line 19. * “… used. Sex…”

Line 23. 2/3 and 1/3 better written out as text.

Line 26. First mention of “lvl”, so best to write long form.

Line 60. * “… to evolve, two new…”

Line 64. * “… maculation, the material…”

Line 88. * “… three lighting treatments (direct, mixed, and diffuse), and then….” Also, be consistent the capitalisation of these treatments – the main text includes these in full capitals.

Line 112. The start of this sentence doesn’t seem to make sense.

Line 121. *four
