# Supplementary material for: Shining a light on camouflage evolution: Using genetic algorithms to determine the effects of geometry and lighting on optimal camouflage
Source: PLoS One. 2026 Apr 29;21(4):e0346231. doi: 10.1371/journal.pone.0346231 (PMC13127904; doi:10.1371/journal.pone.0346231)
Supplement: Attachment — Submitted filename: Reviewer Comments.docx [file pone.0346231.s006.docx]

**Dear Editor**
We would like to thank you for the opportunity to revise and resubmit our manuscript and we would like to apologise for the long delay to our resubmission. One of our co-authors retired during the November and December period, while another oversaw their farewell conference.

We hope that we have been able to address the issues of clarity and have responded accordingly to our reviewers, reducing the amount of technical jargon and providing clarifications to the text where necessary in order to make our results and predictions easier to interpret.

While we understand the request to construct an infographic, and we attempted number of different iterations, we found that they overlapped too much with existing figures or were too large to be clear to understand. One of the main reasons for this being that the summary table at the start did not include mixed lighting as there was little prior literature to make predictions from with the exception of countershading. Meanwhile duplicating the number of columns to simultaneously show predictions and outcomes was too large to fit on a single page and still include a break down of why the prediction was met / failed.

**Reviewer 1**

Reviewer #1: This study used human participants playing an online object finding game, in combination with genetic algorithms, which allow object pattern and color to evolve, to test the effect of light environment and habitat complexity on the evolution of camouflage. They found that light environment does indeed have considerable effects on the optimal camouflage coloration and pattern, particularly in complex habitats, as shadows add to background complexity. This is a clever and interesting study that provides experimental evidence relevant to multiple theories on the evolution of camouflage.

I do however have a couple of suggestions, as well as some questions for the authors.
The paper was sometimes difficult to follow – although I must stress I do not think this is the fault of the authors, rather it is simply because it contains a lot of information, with multiple predictions and results. I did appreciate Table 1 as it gave a clear overview of the predictions – I wonder however if a table like this could be repeated, including which predictions were proven true/false. Otherwise one has to go back and forth between the table and the results/discussion to get a clear overview of the findings.

Response: We attempted and trialled several iterations of infographics but we found them to be too large, dense (original infographic did not included mixed lighting) and overly overlapping with the existing figures. We instead opted to make the existing figures a bit clearer.

R1.02. Technical language was used in the abstract which could be an issue for the broader readership (this also contrasted with the introduction, which for the most part was much more tailored towards a general audience). I would suggest making the abstract a little less technical.
Could repeat players getting better at the game account for the populations that didn’t show improved fitness over the generations? What information do you have on individual players in the game and are they limited in how many times they can participate? I saw that players could only play one population per generation – but how many players played all generations? This aspect was not discussed at all, and while I know these type of games are now very well-accepted and have been used in a lot of studies I still think it’s worth a couple of lines about your “predator” population.

Response: Unfortunately, one limitation of running the game online and using anonymous players was that people could indeed play the experiment multiple times. The only information for each play that was gathered was the “survival_time” for each slide. While it is feasible that improvement in individual player performance could have accounted for some of the failure to improve capture time it would not explain the fact that failure to improve was significantly more likely in less complex habitats or the failure to improve in camouflage within these populations as each generation was independently ranked.

We have now amended “(d) Online experiment design:” to reference the fact that players could play multiple times

L198 To change the target from matt to glossy, the RGB values of the glossy targets were pasted with addition (see S1_File). <- I did not understand this sentence.

Response: we have updated the sentence as follows “To change the target from matt to glossy, the image of the glossy target was combined with the patterned target using image addition. To provide a range of glossiness values, the image of the glossy target was multiplied by a genetically controlled alpha value (see S1_File for more details).

L284 I also found this hard to follow – perhaps due to a lack of explanation of what the techniques used actually do: target edge disruption was measured using gabrat of the target’s surrounding in the luminance channel, with the default sigma of 3, against its original background (58). <- I think many people (including myself) don’t really know what gabrat does.

Response: we have now provided a brief description of gabrat. “Gabrat works by quantifying the ratio of “false” edges to “coherent” edges around the object's outline and has been shown to predict search time for camouflaged disruptive prey by humans.”

L325 is this sentence supposed to trail off…?

Repsonse: apologies, yes it was meant to trail off to indicate additional pieces of code e.g. the dataframe. However, we should have closed the bracket. It is now “lmm (phenotypemetric ~ N-Gen * Light * 3D + (1|Habitat) + (1|Population) … ).”

L325 – I don’t understand why you included if the slope was negative as a random factor – what is this doing exactly?

Response: We have updated the sentence as follows. “To account for instances where survival time failed to improve, a binary variable for whether the population had a negative slope for survival time with n-generations was used as a random factor. “

**Reviewer 2**

Reviewer #2: The authors present a comprehensive computational approach to assess the efficacy of camouflage strategies in conjunction with changes in light and habitat geometry. Given the numerousness and diversity of interactions between these ecological features in the wild, as well as the difficulty in recreating and standardising these interactions in a non-computational experimental set-up, I agree with the claim by the authors that their approach is the first and most optimal solution for addressing this question. The paper therefore presents a very useful foundation for further camouflage investigation and will be of interest to many readers. I have no major concerns with the content, though I believe the clarity of the writing and punctuation needs to be improved at times (outlined in my minor comments). While I understand that the scale of this study and the nature of the question generates a multitude of different statistical comparisons and outputs, I do also fear that the results section may be slightly overwhelming to some readers. I therefore wonder if these results can be summarised with clearer language (i.e., not using model terms) and infographics, as demonstrated in the predictions table (which was very useful for visualisation).

Minor comments
Line 51. Different referencing style for “(Matchette et al., 2018, 2019)”

Response: Corrected, it had been accidentally unlinked from the citation manager.

Line 52. Consider rephrasing to “… maintain effective camouflage, as well as to maximise salience when signalling”

Response: rephrased as requested.

Line 56. Consider rephrasing to “… what should camouflage, which is specialised to either fixed or variable lighting environments, look like”

Response: rephrased as requested

Line 58. * “On a cloudless day, when the habitat is open, …”

Response: Modified as requested.

Line 103. * “For example, comparisons of…”

Response: Modified as requested.

Line 104. There is a big emphasis on countershading within this paper, which is reasonable given its efficacy naturally depends on the light environment. However, the set-up (i.e., viewing targets from a birds-eye-view) is unrepresentative of the most common natural forms of countershading, where the effect is most pronounced when viewed laterally (with the effect representing background matching when viewed from above or below). I agree that, here, a countershading effect may evolve (if direct lighting is present) and that this is informative, but the conditions and context for this emergence here are less naturalistic and I think this needs to be caveated for the reader.

Response: We agree that this is a potential caveat of our paper and we reference this in lines 570-572 “It is possible that countershading is still beneficial when viewed from the side where the background appearance differs in visible structure.”. To further highlight this point we have modified the line to emphasise that many camouflaged animals will be viewed from the side “In particular, our targets were viewed from above, rather than from the horizon, as will be the case for many animals such as the ungulates previously studied. Differences in the orientation of shadows within the scene at this orientation may alter which countershading patterns most effectively conceal prey”

Line 110. * “… enhancement patterns, which pictorially mimic shadows that form at the edges of objects from direct lighting, are harder…”

Response: Modified as requested.

Line 119. * “…thought to be one factor…”

Response: Modified as requested.

Line 121. * “change select for background matching…”

Response: We aren’t certain if this recommendation is correct. The sentence has been modified as follows: “But does the stability of information change the intensity of selection for background matching within these channels? “

Line 126. * “e.g., glossiness (44)”

Response: Added missing comma

Line 128. Consider restructuring the sentence introducing the predictions table.

Response: We have revised the table legend accordingly.
**Table 1.** A summary of the predicted effects of lighting condition, habitat 3D variation and the interaction between direct lighting and 3D variation on the appearance of all animal patterns. Upwards arrows indicate a positive effect on the phenotypic feature, e.g., increased luminance or directionality, while downwards arrows indicate a negative effect; a horizontal line indicates no predicted effect. Larger arrows indicate that increased variation increases the effect size of direct lighting, while smaller arrows indicate a reduction in effect size. For each prediction, the justification and relevant references are provided. CIE L*, a* and b* refer to the mean values of the opponent colour channels used by the CIELAB perceptual colour space (50). These channels are L* (luminance, low = dark, high =light), a* (red-green, low = green, high = red) and b* (blue-yellow, low = blue, high = yellow).
Line 138. I’d move the reference for CamoEvo to directly next to it, then adjust to “which we used to disentangle…”

Response: Adjusted accordingly.

Response: Modified as requested.
Line 140. Be consistent with capitalisation for every mention of CamoEvo.

Response: We’ve ensured all instances now show “CamoEvo”.

Line 148. * “… by lighting, such as…”

Response: Modified as requested.

Line 149. This line is a repeat of the earlier statement; consider removing this or the earlier version.

Response: We are uncertain as to what exactly this refers to.

Line 153. * “…directionality, while downwards…”

Response: Modified as requested.

Line 154. * “… a negative effect; horizontal lines indicate no effect.”

Response: Modified to “; a horizontal line indicates no predicted effect”.

Line 156. The phrase “refer to the axes widely used CIELAB perceptual colour space” needs rewording for increased clarity.

Response: Modified to “CIE L*, a* and b* refer to the mean values of the opponent colour channels used by the CIELAB perceptual colour space (50). These channels are L* (luminance, low = dark, high =light), a* (red-green, low = green, high = red) and b* (blue-yellow, low = blue, high = yellow).”

Line 168. Do you have some quantification of how much the intensity of light varied between direct light treatments across habitats?

Response: Unfortunately, we did not take any additional light intensity measures, e.g. the ambient irradiance of the target location. While it would have been good to include intensity as an additional random effect, the core goal of this study was to look at the influence of the directionality of ambient light rather intensity. Variation in time of day and surrounding 3D structures would have created noise in light intensity, directionality (e.g. lower sun = greater directionality of light), etc. Despite this noise, the overall average effect of directionality and geometry was maintained.

Line 172. From here on, the use of “Fig” should be adjusted to “Fig.”

Response: Modified as requested.

Line 174. Did you have some procedure when choosing which habitat patch to photograph (and therefore where the target was placed)?

Response: Due to weather restrictions (limited availability of <10% cloud coer), restrictions to pre-mapping the sites due to COVID, and the inability to place the photography tent in some locations we weren’t able to pre-map the site areas. Habitat patches were visually identified by possessing similar background features and weren’t allowed to overlap, i.e. the distance from the previous location had to be greater than the dimensions of the photography tent (1.5 m).

Line 183. An example would be helpful for “other tall objects”

Response: Rephrased to “3D variation featured vegetation and other elevated surfaces, e.g., large stones.”

Line 193. * “glossiness of the background pattern.”

Response: Rephrased as requested.

Line 193. Consider rephrasing this sentence e.g., “For full details of this system, see the Supplementary Materials and XXX et al. (XX)”

Response: Rephrased as requested.

Line 200. The phrase “were then randomly cropped to create the stimuli” needs more clarity for the reader.

Response: Sentence has been adjusted as follows “Background images with rendered patterns were then cropped with the target position randomly offset from the centre to ensure the target wasn’t always in the same position (S1_File). Each cropped image was 1904 ×1488 px, and the target button had a diameter of 80 px.”

Line 202. Be consistent with language (e.g., “target” or “target button”), or more explicitly introduce the “button”.

Response: Button has now been introduced in (a) Visual Scenes “Within each scene, a 3D-printed 30 mm diameter button-shaped target painted grey with a matt 8% reflectance paint was placed at the centre.”

Line 208. * “… (DIFFUSE), and then a mixed treatment…”

Response: Rephrased as requested.

Line 214. * “.. the previously mentioned stimuli.”

Response: Rephrased as requested.

Line 215. What was the source for the existing javascript?

Response: The sentence has been re-written to make it clear that the game was modified from the following experiment: “An online computer-based search game ( <https://CamoEvo.visual-ecology.com/> ) was created by modifying JavaScript used for Troscianko et al., 2020 ‘s crab camouflage experiment. “

Line 216. * “With this set-up, the goal was to use humans…”

Response: Rephrased as requested.

Line 219. * “capturing the targets, one by one, as fast as they could.”

Response: Rephrased as requested.

Line 223. * “… displayed in a random order.”

Response: Rephrased as requested.

Lines 230-235. I think this section needs a little more clarity. So, if I have this right, ‘response time’ is the time between the start of the trial and the time at which point the cursor breaks the circle, whereas capture time comprises the response time plus the time from circle to the target. It would be good to get a sense of the number of trials that capture time and response time are used respectively. If capture time is used in extreme cases (i.e., delayed or unreasonably fast) as well as for all touch screen interactions (again, an n would be useful), then why not just use capture time throughout (for consistency)? The legend for Figure 1 also states that the selection of targets was based on the time taken to click on the targets, which wouldn’t have been the case for “response time” instances (unless I’ve misunderstood).

Response: We have rephrased figure 1 to make it clear that survival time was used. Yes, we agree with the reviewer here and this information we have outputted prior for the CamoEvo toolbox. Unfortunately, the online game was run with an earlier version of CamoEvo before its final release and so did not implement separate outputs for survival, response, and capture time for each generation. Only the ID name of the target and its fitness (survival time). There was supposed to be an Output.txt directory which would save all of this information, in addition to the fitness tables for each population and generation. However, the file froze at generation 3 for unknown reasons.

Line 234. Be more explicit that the measure is a proxy for survival time (rather than “dubbed”)

Response: Rephrased as follows: “This measure was used as a proxy for survival time, following the same protocol successfully used for the CamoEvo toolbox (46). “

Line 237. Wrongly formatted reference.

Response: Citation has been re-linked to citation manager and corrected.

Line 246. Is “(Gen)” needed here?

Response: Corrected to “Gen 0”

Line 302. * “… Dev=1, while lighting treatment was encoded as…”

Response: Rephrased as requested.

Lines 302 and beyond. Be consistent with “lighting treatment” – I think it makes it easier to follow.

Response: Rephrased as requested.

Line 312. Consider introducing the formula as: “Using lme4 nomenclature, the models were as follows:”. Also, there is no need for the end of this sentence to still be in italics, and can be isolated as a new sentence.

Response: Rephrased as requested.

Line 315. * “… geometry (i.e., pattern contrast…”

Response: Rephrased as requested.

Line 316. * “… glossiness), we first…”

Response: Rephrased as requested.

Line 327. I think the response measures that have been transformed need to be explicitly mentioned (for replicability’s sake)

Line 332. * “… tables are provided in the Supplementary Materials (S2_File).”

Response: Rephrased as requested.

Line 336-338. Consider rephrasing this sentence to “To assess whether targets become more camouflaged, we measured the change in survival time (i.e., our fitness proxy) as a function of generation number (Fig. 2).” For the layman (and general readability), I’d also stick to phrases like “generation number” rather than “n-generation” throughout.

Response: Rephrased as requested with n-generations replaced with generation number.

Line 337. * “… improved generation number (via a model not using…”

Response: Rephrased as requested.

Line 342. Add number of replicates here i.e., “… increase in fitness (n = 3) or decreased in fitness with generation number (n = 12).”

Response: Rephrased as requested but to N=6 and N=12 as we realised the number had been mis-reported.

Line 344. Consider rephrasing to “Populations where the generation number lowered survival time were excluded from subsequent analyses, with the exception of the habitat analysis.”

Response: Rephrased as follows: “Populations where the generation number lowered survival time were excluded from subsequent analyses, with the exception of the habitat analysis below.”

Line 347. Best to choose either variation or complexity (rather than use a forward slash).

Response: Rephrased to just complexity.

Line 360. Sentence ends abruptly after “which”.

Rephrased to: “Whereas, for improvement in fitness with generation number, it was background B variance which had the largest effect”

Line 365. *than

Response: Rephrased as requested.

Line 373. *effect

Line 392-395. Why Ai if only one panel (same applies to later figures)? Bi needs to have slightly more information i.e., including information from the key. The start of the description for Bii is also needs to be made more clear. Change from “pattern match” to “pattern difference” (as this is what is in the figure)

Response: Removed Ai for both figures 2 and 3 and we have updated the text of figure 2 to highlight the legend and make clearer the link between the camouflage metrics and fitness.

Line 451. * “…for all substrates under both mixed and direct lighting, except gravel under the direct lighting condition”.

Response: Corrected as requested.

Line 453. * high

Response: Corrected as requested.

Line 499. *see

Response: Corrected to “see Supplementary Material, S1_File”

Line 514. * “… of Merilaita’s (3) oft-cited model…”

Response: rephrased as requested.

Line 525. * “… critical relationships between lighting and camouflage with habitat geometry.” (Then delete the rest of that sentence)

Response: rephrased as requested.

Line 545. This paper does not directly test the military aspect, so an additional reference is needed here.

Response: Sentence has been restructured and now includes a reference to “US Company of Engineers. 1941. Notes on modern camouflage. Fort Belvoir, VI, USA: Camouflage Section, Engineer Board.”

Line 563. * “… and, by extension, make…”

Response: rephrased as requested.

Line 574. * “… has yet…”

Response: rephrased as requested.

Line 577. Is maculation the best word here? This term has been used a few times in the paper, and I think it probably needs to be more clearly defined at its first reference i.e., my image of this word is spots, hence why “directional striped ‘spots’” are hard to visualise

Response: we used the term maculation as it both references the marking elements of the target as opposed to the counter-shaded background and it aligns with the terminology used to distinguish these regions in CamoEvo. A maculation simply refers to as a marking and does not necessarily mean spotted. Though its more archaic use is just for spotted. To avoid confusion, however, we have replaced maculation with marking in the main text and supplement.

Line 588. * “… turn be aligned with them.”

Response: rephrased as requested.

Line 609. Consider rephrasing to “We predict that simple ecological and behavioural differences…”, mainly because these haven’t been tested.

Response: rephrased as requested.

Supplementary: S1_File
Line 19. * “… used. Sex…”
Line 23. 2/3 and 1/3 better written out as text.
Line 26. First mention of “lvl”, so best to write long form.
Line 60. * “… to evolve, two new…”
Line 64. * “… maculation, the material…”
Line 88. * “… three lighting treatments (direct, mixed, and diffuse), and then….” Also, be consistent the capitalisation of these treatments – the main text includes these in full capitals.
Line 112. The start of this sentence doesn’t seem to make sense.
Line 121. *four

Response: Adjusted all as suggested.
